# Heritable Change Caused by Transient Transcription Errors

**DOI:** 10.1371/journal.pgen.1003595

**Published:** 2013-06-27

**Authors:** Alasdair J. E. Gordon, Dominik Satory, Jennifer A. Halliday, Christophe Herman

**Affiliations:** 1 Department of Molecular and Human Genetics, Baylor College of Medicine, Houston, Texas, United States of America; 2 Department of Molecular Virology and Microbiology, Baylor College of Medicine, Houston, Texas, United States of America; Universidad de Sevilla, Spain

## Abstract

Transmission of cellular identity relies on the faithful transfer of information from the mother to the daughter cell. This process includes accurate replication of the DNA, but also the correct propagation of regulatory programs responsible for cellular identity. Errors in DNA replication (mutations) and protein conformation (prions) can trigger stable phenotypic changes and cause human disease, yet the ability of transient transcriptional errors to produce heritable phenotypic change (‘epimutations’) remains an open question. Here, we demonstrate that transcriptional errors made specifically in the mRNA encoding a transcription factor can promote heritable phenotypic change by reprogramming a transcriptional network, without altering DNA. We have harnessed the classical bistable switch in the *lac* operon, a memory-module, to capture the consequences of transient transcription errors in living *Escherichia coli* cells. We engineered an error-prone transcription sequence (A_9_ run) in the gene encoding the *lac* repressor and show that this ‘slippery’ sequence directly increases epigenetic switching, not mutation in the cell population. Therefore, one altered transcript within a multi-generational series of many error-free transcripts can cause long-term phenotypic consequences. Thus, like DNA mutations, transcriptional epimutations can instigate heritable changes that increase phenotypic diversity, which drives both evolution and disease.

## Author Summary

The transfer of information from cell to cell is crucial for preserving cellular identity. Errors in DNA, RNA or protein synthesis are universal and inevitable. While errors in DNA synthesis can produce heritable mutations that can change the phenotype of the cell, errors in transcription are considered transient with no long-term consequence since mRNA is short-lived and the encoded erroneous proteins are eventually degraded. Previously, using the *lac* operon system, a paradigm of gene regulation with a bistable behavior, we showed that an overall reduction of transcription fidelity in the cell triggers cellular identity change. However, a direct demonstration that errors in the mRNA encoding the regulator of the bistable switch were indeed responsible for triggering the phenotypic change rather than indirect effects of transcription infidelity was still lacking. Here, we show directly that transcription errors provoking frameshifting in a coding sequence of a regulator involved in a genetic program, can promote change in cellular identity. Therefore, this is a direct genetic demonstration that a transient error in a discrete transcript can produce a heritable change in phenotype. Like protein conformation changes in prions and mutation in DNA, we postulate that mRNA errors will have the same implications for disease and evolution.

## Introduction

Stable phenotypic change is mostly associated with DNA alteration [1], the hardware of the cell, but rarely as the consequence of errors in the transmission of cellular genetic programs, the software of the cell [2], [3]. Transcription factors play a critical role in establishing cellular programs and heritable cellular identity [4], as elegantly shown by somatic cell nuclear transfer [5] and more recently reprogramming of differentiated cells into pluripotent cells [6]. Among cellular genetics programs, bistable gene networks play an important role in cellular differentiation and identity by allowing expression of multiple stable and heritable phenotypes from one single genome [7]. Examples of bistable systems include the *E. coli* lactose-operon-repressor system [8], the lambda bacteriophage lysis–lysogeny switch [9], the genetic toggle switch in bacteria [10] and human cells [11], phosphate response in yeast [12], cellular signal transduction pathways in *Xenopus* [13], HIV virus development [14], and the “restriction point”: the critical switch by which mammalian cells commit to proliferation and become independent of growth stimulation in cancer [15]. Recent studies on single cell genealogy analysis have revealed that heritable stochastic change can occur by dysregulation of bistable regulatory networks [16], [17]. This phenotypic switching has been associated with infrequent large bursts in transcription, generated by the stochastic dissociation of a transcription factor from its DNA regulatory site and resulting in a change of expression pattern [17].

Our previous studies suggested that transcription infidelity could contribute to stochastic heritable phenotypic change in a bistable gene network [18]. We showed that the removal of transcription fidelity factors in the cell, GreA and GreB (functional analogs of eukaryotic TFIIS), triggers heritable stochastic change [18]. Therefore, we proposed that an overall decrease in transcription fidelity affecting all nascent transcripts in the cell can increase stochastic switching in this system by altering the quality of the transcription factor involved in the bistable switch. However, these transcription fidelity factors also have other functions in transcription initiation and elongation [19]. In addition, a global decrease in transcription fidelity may indirectly trigger phenotypic switching by globally impacting the physiology of the cell, instead of directly altering the transcription factor mRNA [20], [21]. Due to the unstable nature of mRNA, the direct capture of the erroneous mRNA responsible for the phenotypic switch is not currently possible since by the time the cell exhibits the new phenotype, the initial erroneous mRNA will have been degraded. Hence, we have now developed a novel genetic approach to show that mRNA errors specifically in a transcription factor involved in a bistable switch can directly trigger heritable phenotypic change in a clonal cell lineage.

In *E. coli*, the *lac* operon has been shown to behave like a bistable switch and is considered a model system of gene regulation. The *lac* operon comprises a positive feedback loop that allows bistability under a specific concentration of inducer, thio-methylgalactoside (TMG), known as the maintenance concentration [8]. The lactose permease protein (encoded by the *lacY* gene) transports its own inducer, which in turn activates permease synthesis by derepressing its operon *via* inactivation of the *lac* repressor. Due to this autocatalytic positive feedback loop the *lac* operon exhibits two persistent and heritable expression states depending on the cellular history [8], [18], [22]. In the presence of the maintenance level of inducer, cells with permease will stay induced (ON) and cells without permease will stay uninduced (OFF) but will have a probability of switching ON. In their classic experiment, Novick and Weiner showed the persistence of the two heritable expression states for over 180 generations in a chemostat [8].

To directly test that transcription errors in the mRNA of a transcription regulator can promote phenotypic change, we engineered a transcriptional error-prone sequence into the *lacI* repressor gene, which dictates the fate of a heritable ON/OFF epigenetic switch in the *lac* operon. If our model that transcription errors cause epigenetic switching is correct, we predict that this engineered transcription error-prone sequence would lead to increased epigenetic ON-switching in the bistable *lac* system.

## Results and Discussion

### Engineering an Error-prone Sequence in the *lacI* Transcript

**Figure 1:**
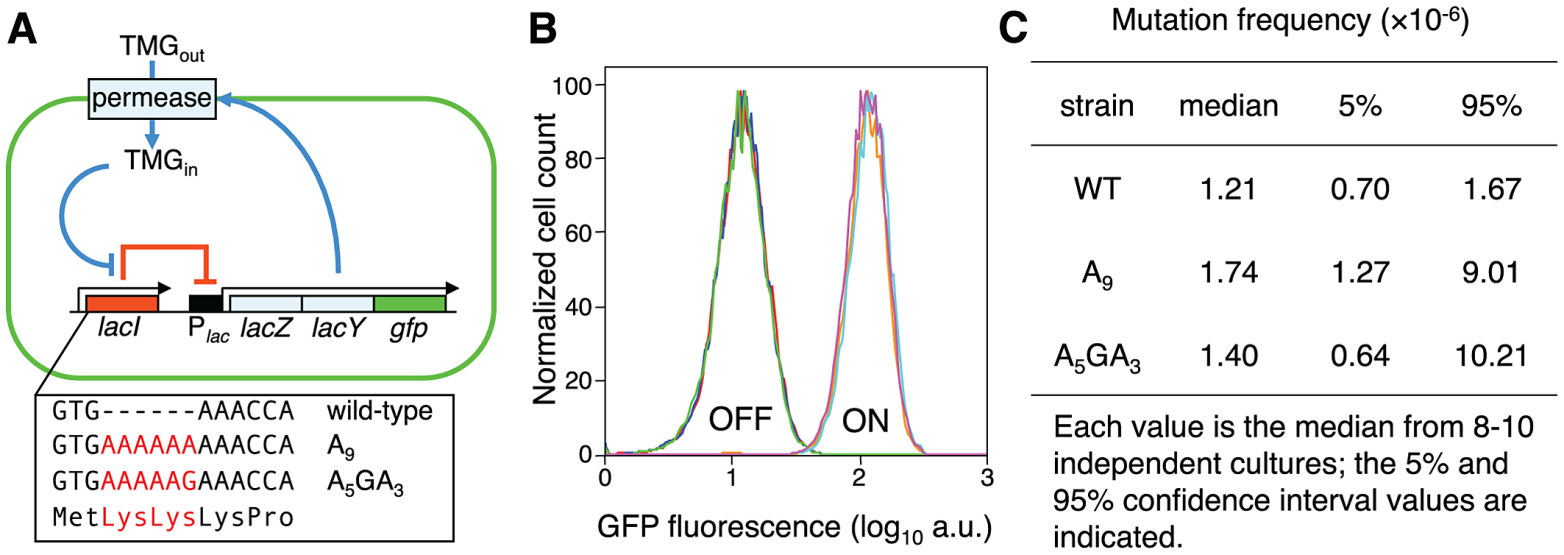
Novel system to study the consequences of error-prone transcription sequences. (A) Under maintenance conditions, the *lac* operon is OFF (indicated by the solid red line) and the inducer TMG remains extracellular; stochastic events that lead to a transient derepression of the *lac* operon will initiate an autocatalytic positive-feedback response (indicated by solid blue lines). The box highlights the first three codons of the wild-type *lac* repressor gene and the Lys-Lys additions encoded by the A_9_ and A_5_GA_3_ *lacI* alleles (in red). (B) The *lacI* A_9_ and A_5_GA_3_ Lys-Lys N-terminal addition alleles encode functional *lac* repressors. Representative flow cytometry analyses measuring GFP fluorescence of OFF and ON populations of wild-type (red, brown), A_9_ (blue, light blue) and A_5_GA_3_ (green, violet) *lacI* cells produce identical histograms; 10^4^ cells of each strain were interrogated. (C) Forward *lacI* ^+^→*lacI* ^−^ mutation frequencies. No significant difference in mutation frequency between the A_9_ and A_5_GA_3_ strains is observed (Mann-Whitney Rank Sum Test, *p* = 0.23). The wild-type strain is added for comparison.

To directly look at the consequence of transcription errors in *lacI* mRNA, we elongated the native A_3_ sequence at the 5′ end of the chromosomal *lacI* gene to an A_9_ run or an A_5_GA_3_ control sequence (Figure 1A; Figure S4A) and thereby created a novel *lac* repressor that has two additional N-terminal appended Lys residues. Monotonous runs of adenines in DNA are hotspots for RNA polymerase slippage events during transcription in *E. coli* [23]–[26], *Thermus thermophilus* [27], yeast [25], [28] and human cells [29]–[31]. During transcription, the **∼**8 bp hybrid between the nascent RNA chain and the DNA template maintains the proper register of the RNA transcript [32]. With a T_9_ run in the template (and an A_9_ run in the coding strand, as is found in our study), the growing chain with eight or nine A residues can dissociate from the template and realign out of frame, while still maintaining the required 8 bp RNA∶DNA hybrid. Therefore, the minimum length of the T run to promote transcriptional slippage is T_9_ [24], [27], [28]. In a wild-type gene, this transcriptional slippage results in the addition or deletion of a ribonucleotide in the A run in the transcript, resulting in a shift in the open reading frame and producing a burst of nonfunctional truncated protein.

**Figure 2:**
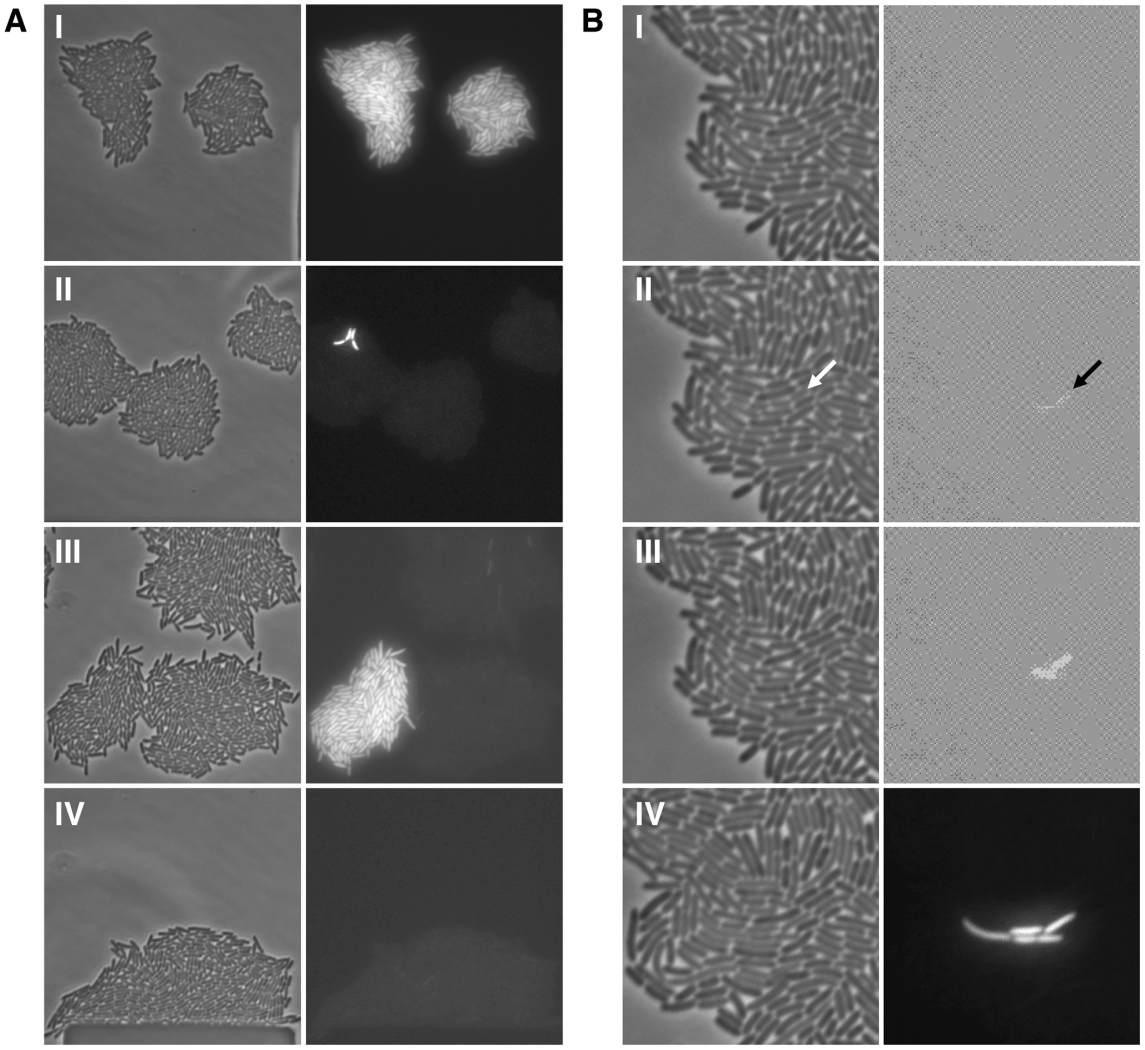
Bistability, hysteresis and stochastic switching in the *lac* system. (A) Single *lacI* A_9_ cells in minimal succinate media ± maintenance TMG were grown into microcolonies in a microfluidic flow chamber (approximately 100 cell divisions per microcolony originating from a single cell; number of divisions equals final number of cells in a microcolony minus 1). Comparison of bright field images (panel series on the left) with GFP fluorescence images (panel series on the right) allows clear distinction between OFF and ON cells in the microcolony. (I) panels show microcolonies that arose from single ON cells that were subsequently grown in the presence of maintenance level TMG; (II) panels show mirocolonies that arose from single OFF cells that were subsequently grown in the presence of maintenance level TMG; (III) panels show mirocolonies that arose from single OFF and single ON cells that were subsequently grown in the presence of maintenance level TMG; (IV) panels show a mirocolony that arose from ON cells that were subsequently grown in the absence of maintenance level TMG. Exposure to fluorescence illumination was 3000 ms. (B) A single *lacI* A_9_ cell in minimal succinate media+maintenance TMG was grown into a microcolony in a microfluidic flow chamber and monitored by time-lapse fluorescence microscopy. Presented here are four still images from a full time series of images (available as Movie S1; images shown correspond to frames 25, 28, 30, 39). Comparison of bright field images (panel series on the left) with GFP fluorescence images (panel series on the right) allows distinction between OFF and ON cells in the microcolony. (I) all cells in the microcolony are OFF (100 ms exposure to fluorescence illumination); (II) a recently divided cell is just becoming ON indicated by the arrow (100 ms exposure to fluorescence illumination); (III) the now separated cells have become ON (100 ms exposure to fluorescence illumination); (IV) further cell division has occurred in the microcolony creating one lineage of ON cells amongst many other lineages of OFF cells (3000 ms exposure to fluorescence illumination). The 100 ms exposure time images were over-exposed using the ColorSync Utility to observe the faint fluorescence signal.

**Figure 3:**
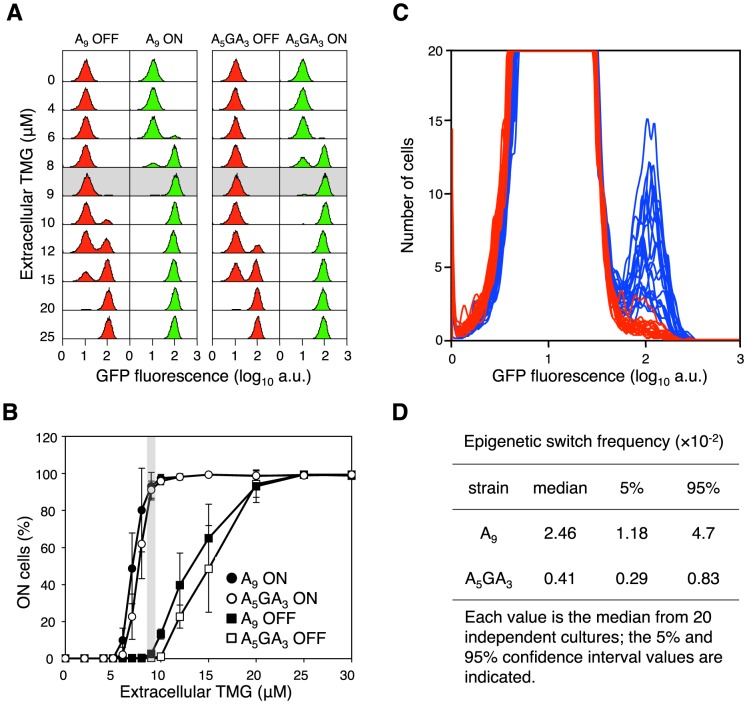
The error-prone A_9_ run in the *lacI* transcript increases stochastic phenotypic switching. (A) Representative flow cytometry GFP fluorescence histogram series of A_9_ and A_5_GA_3_ *lacI* cells that were originally ON (green histograms) or OFF (red histograms) were sub-cultured and grown in media containing various concentrations of TMG indicated on the vertical axis (10^4^ cells interrogated). Below 5 µM TMG and above 20 µM TMG, the previous history of the cell (ON or OFF) does not affect the current state of the cell; between these TMG concentrations the system exhibits hysteresis. The shaded area highlights the maintenance concentration of 9 µM TMG for these strains. (B) Cells that were originally ON or OFF were sub-cultured and grown in media containing various concentrations of TMG, as above. Each value is the average ± SD from 5 to 15 independent cultures. The shaded area highlights the maintenance concentration of 9 µM TMG for these strains. (C) OFF A_5_GA_3_ *lacI* cells (red histograms) and A_9_ *lacI* cells (blue histograms) were diluted and grown in media containing 9 µM TMG. After 42 h growth, flow cytometry was performed to determine the frequency of epigenetically ON cells in 20 independent cultures of each strain; the A_5_GA_3_ histograms are superimposed over the A_9_ histograms (10^4^ cells interrogated). (D) The A_9_ epigenetic-switch frequency is significantly increased over the A_5_GA_3_ value (Mann-Whitney Rank Sum Test, *p*<0.001).

Single cell analysis by flow cytometry shows that the GFP fluorescence histograms produced from OFF and ON populations of wild-type, A_9_ and A_5_GA_3_ repressor strains are identical demonstrating that the engineered Lys-Lys addition does not perturb repressor function (Figure 1B). The altered repressors still recognize and bind the *lac* operator, negatively regulate the *lac* operon and remain responsive to TMG, which binds to the structurally distinct C-terminal core domain and induces an allosteric transition in *lac* repressor so that it no longer binds to the *lac* operator. Moreover, all three repressor allele strains (wild-type, A_9_ and A_5_GA_3_) exhibit the same basal level of β-galactosidase activity in OFF populations indicating that the altered repressors bind as well as the wild-type repressor to the *lac* operator (Figure S4B). In addition, there is no difference in the spontaneous *lacI* ^+^→*lacI* ^−^ mutation frequency for these two altered *lacI* alleles (Figure 1C; Text S1). Finally, the A_9_ and A_5_GA_3_ repressor/*lac* operon gene networks also exhibit bistability and hysteresis as has been shown for the native *lac* system (Figures 2, 3A,B; Figure S4C) [8], [18], [22]. To interrogate a larger number of cells, we used flow cytometry (Figure S1) and validated this method by showing that the frequencies of switching in diverse genetic backgrounds are similar to what we previously published (Figure S2). Thus, the discrimination afforded by flow cytometry analysis of GFP fluorescence between ON and OFF cells for the wild-type and variant *lacI* alleles is sensitive and sufficient to monitor stochastic switching.

### Transcriptional Slippage in the *lacI* Gene Increases Epigenetic Stochastic Switching

To determine the proportion of cells that are ON, we used the green fluorescent protein gene integrated within the *lac* operon (Figure 1A) [18]. During growth of OFF cells in a maintenance concentration of TMG, if a cell suffers a stochastic event leading to derepression of the *lac* operon, *e.g.* a transcription slippage error in *lacI*, the *lac* operon will be transiently derepressed triggering permease synthesis and activation of the autocatalytic positive-feedback loop, resulting in green fluorescent cells [18]. As a result, the OFF state will transition to the ON state and be heritably maintained in the following generations, mimicking *lacI* mutation in this system (*i.e*. transient stochastic events in information transfer can have heritable phenotypic consequences; Figure 2A,B) [8], [18]. We calculate the switch frequency as number of ON cells over the total number of cells interrogated, following the convention used in determining *lacI* ^−^ mutation frequencies [33]. The observed ON switch frequency is therefore dependent on both the number of switch events that have occurred and the number of generations after a discrete switch event has occurred, as in a classical fluctuation test; our experiments run for ∼28 bacterial generations (see Materials and Methods).

Between the two strains harbouring A_9_ and A_5_GA_3_ alleles, we can assess how slippery transcription sequences affect stochastic switching in the bistable *lac* system. We observe a 6 to 12-fold increase in switch frequency (Mann-Whitney Rank Sum Test, *p*<0.001) for the A_9_ construct compared with the A_5_GA_3_ control in a wild-type background at the maintenance concentration of TMG (Figure 3C,D; Figure S5). This A_9_ epigenetic switch frequency is fully 10,000 times greater than the genetic *lacI* ^−^ mutation frequency demonstrating that it is not mutation underlying the observed stochastic switching.

**Figure 4:**
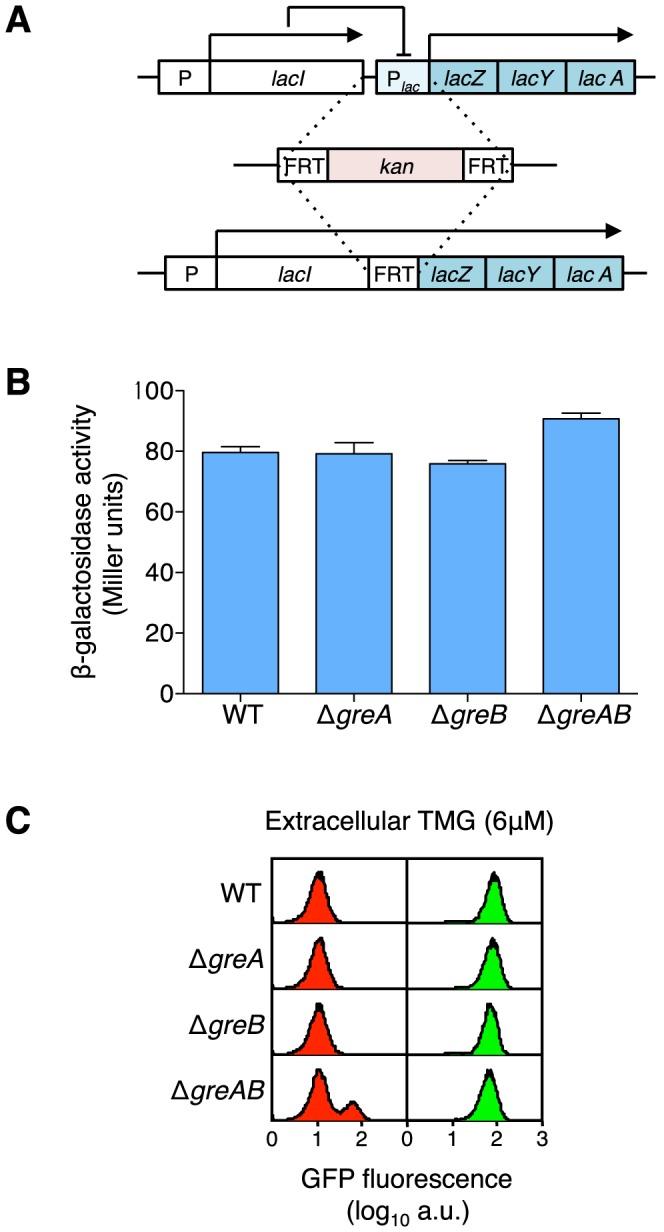
Creation of a *lacIZYA* operon fusion to assess the levels of gene expression from the *lacI* gene promoter. (A) An operon fusion was created by first inserting a kanamycin cassette from pKD4 (Table S3) into the intervening region between *lacI* and *lacZ* and then, *via* a flippase reaction, removing most of *lac* operator *O_3_*, the complete *lac* promoter and *lac* operator *O_1_*. Therefore, the *lacIZYA* fusion transcript is under the expression of the weakly constitutive *lacI* promoter with no interference from any *lac* repressor binding (*lac* repressor does not negatively regulate *lac* expression through *O_2_* alone) [60]. The complete sequence of the intervening region from the TGA stop codon of the *lacI* gene to the ATG start codon of the *lacZ* gene is shown in Figure S3. (B) Expression levels of the *lacIZYA* operon fusion strains are equivalent regardless of GreA, GreB or GreAB status indicating the absence of any or all Gre functions does not influence overall *lac* expression levels. Cells were grown in minimal A salts plus glucose and β-galactosidase levels were determined by the method of Miller [33]; the average ± SD for three independent cultures is shown. To make functional β-galactosidase in this fusion strain the transcription complex must produce at least a 4,271 nt transcript including the *lacI* non-translated leader, the *lacI* gene, the FRT scar sequence and the *lacZ* gene; if the transcript terminates after *lacA*, at the usual *lac* termination site, then the entire transcript will be over 6.2 kb in length. (C) At maintenance level of TMG, the absence of GreA or GreB does not increase stochastic switching over wild-type levels but when both Gre functions are absent a significant increase in stochastic switch frequency is observed [18]. Representative flow cytometry histograms of wild-type, Δ*greA*, Δ*greB* and Δ*greAB* cells that were originally ON (green histograms) or OFF (red histograms) were sub-cultured and grown in media containing a maintenance level of 6 µM TMG. All strains are equally responsive to TMG at this concentration (all ON populations remain ON, *i.e.*, maintain their previous state), but only OFF Δ*greAB* cells exhibit an increased stochastic switching frequency over that observed when wild-type OFF cells were grown at maintenance level of TMG; each histogram represents the interrogation of 10^4^ cells. Therefore, it is not an overall decrease of *lacI* expression that causes the significant increase in stochastic switch frequency in the absence of GreAB.

In addition, the observed increase in phenotypic switch frequency cannot be explained by problems in transcription initiation or early termination for the following reasons. First, native *lacI* mRNA, produced from a weak constitutive promoter, includes a 28 nucleotide (nt) untranslated leader sequence before the GTG initiation codon [34] and the A_9_ run (or the A_5_GA_3_ broken run) should not affect transcription initiation. Since the nascent transcript is 31 nt in length before the A_9_ run is first encountered, transcription of this sequence will be the processive synthesis of RNA as an RNA polymerase-DNA transcription complex and not as an RNA polymerase-promoter initial transcribing complex [35]. Second, aborted transcripts, if they occur from the *lacI* promoter, are also not relevant here; abortive events occur within 8–15 nt from the promoter, far away from the A_9_ (and the A_5_GA_3_) sequence. The absence of GreA function increases transcription abortion [35], however, we see no difference in transcript elongation in *lacI* monitored by a *lacIZYA* fusion in the absence of GreA, GreB or GreA,B function with these constructs whereas we see a significant increase in phenotypic switching in the absence of GreA,B (Figure 4). Third, we show that ±1 frameshifting in the coding sequence of *lacI* does not cause early transcription termination before downstream transcription into the *lac* operon (Figure S6). Together these experiments suggest that perturbations in transcription initiation and early termination are not involved in the increased OFF to ON switching frequency we observe. The net decrease in functional repressor in a cell, due to transcription error, may further require the dilution of ‘old’ wild-type repressors *via* partitioning during cell division to promote stochastic switching [36].

### Transcription not Translation Errors Influence Stochastic Switching

**Figure 5:**
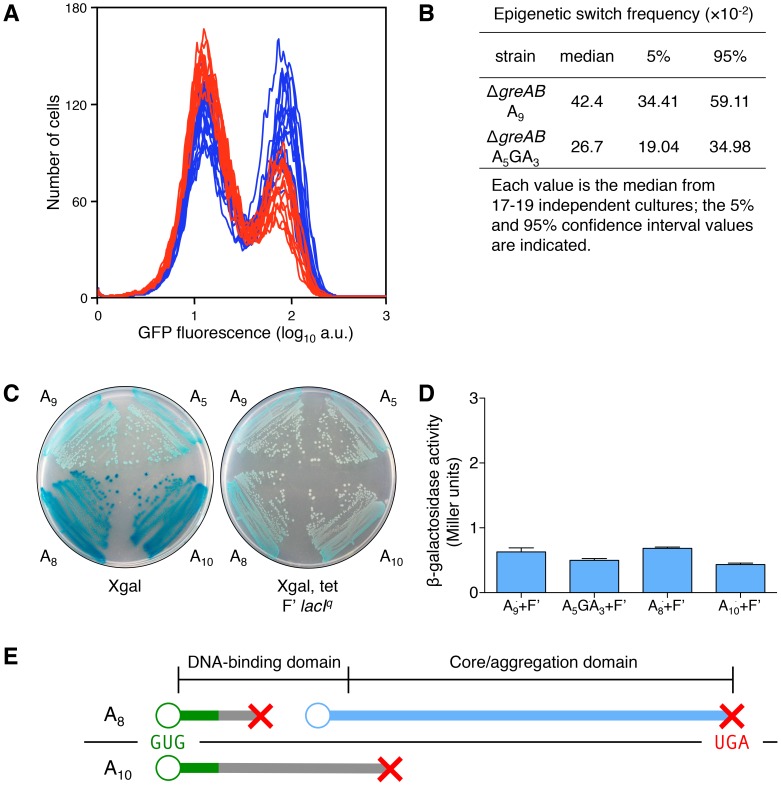
Transcription errors, not translational frameshifting, at the *lacI* A_9_ sequence influences stochastic switching. (A) Stochastic phenotypic switching is significantly increased when the error-prone A_9_ run is in a transcription fidelity-deficient background (Δ*greA* Δ*greB* cells). OFF Δ*greAB* A_5_GA_3_ *lacI* cells (red histograms) and Δ*greAB* A_9_ *lacI* cells (blue histograms) were diluted and grown in media containing 9 µM TMG. After 42 h growth, flow cytometry was performed to determine the frequency of epigenetically ON cells in 17–19 independent cultures of each strain; the histograms from the Δ*greAB* A_5_GA_3_ *lacI* cultures are superimposed over the histograms from the Δ*greAB* A_9_ *lacI* cultures; each histogram represents the interrogation of 10^4^ cells. (B) The median for the Δ*greAB* A_9_ *lacI* strain is significantly different from the Δ*greAB* A_5_GA_3_ *lacI* value (Mann-Whitney Rank Sum Test, *p*<0.001). (C) To model translation frameshifting in our system we have created merodiploids that provide a 10-fold excess of wild-type transcripts over ±1 frameshift transcripts (as modeled by the A_8_ and A_10_ *lacI* alleles). Therefore, the ratio of wild-type transcript over frameshifted transcript, at the level of transcription (10∶1), will be a very conservative approximation of the situation that would arise if during the translation of one A_9_ transcript, one translational frameshift event would occur (20∶1 wild-type sub-units over frameshifted sub-units). The wild-type repressor allele is completely dominant over the frameshifted repressor alleles: left panel, the *lacI* allele strains without the F′; right panel, the *lacI* allele strains with the F′ overproducing wild-type *lacI*. The glucose minimal plates include Xgal (40 µg/ml) and tetracycline (Tet, 12.5 µg/ml), as indicated beneath the plate. Tet is used to maintain the F′ in the cell. (D) Quantitative measurement of the phenotype observed in (C). The level of β-galactosidase in all four strains is comparable and does not exceed 1 Miller unit, which is the basal β-galactosidase of uninduced *E. coli* cells [33]; the average ± SD for three independent cultures is shown. (E) A −1 translational frameshifting event at the A_9_ sequence would cause translation to terminate at codon 4/5 (green line denotes wild-type protein; gray line denotes frameshifted protein; red X denotes translation termination; blue line denotes translation reinitiation protein; the A_9_ transcript is shown as a black line with the GUG start codon in green letters and the UGA stop codon in red letters; the protein domain structure is indicated above the translation products). Therefore, no functional *lac* repressor sub-unit could be produced; however, it has been shown that a dominant-negative sub-unit could be produced by translational reinitiation [61]. Reinitiation could occur at codons 23, 24, 38 or 42 [34], producing repressor sub-units lacking the DNA-binding domain but the core aggregation domain would be intact and able to bind and interfere with wild-type sub-unit function. Therefore, there is the possibility that one −1 translational frameshifting event would not only decrease the net total of repressor sub-units by one, but might also decrease the cell's net *lac* repressors by one, since it has been shown that one dominant-negative sub-unit with three wild-type sub-units may abolish the function of the tetrameric *lac* repressor [42]. A +1 translational frameshifting event at the A_9_ sequence (generating a A_10_ transcript) would cause translation to terminate at codon 83/84 and would therefore only result in the net decrease of one repressor sub-unit in the cell, since no dominant-negative sub-unit can be made this far into the core domain. When the wild-type sub-unit is made at 10-fold the level of ±1 transcription frameshift events (and ±1 translation protein products), the wild-type sub-units dominate and the *lac* operon is repressed (as seen in the Xgal Tet F′ *lacI^q^* plate in (C) and therefore any net decrease by one translation frameshift event is negligible when compared to the net decrease in repressor sub-units due to a transcription error. When a transcription error occurs at the A_9_ sequence, all the nascent *lac* repressor sub-units will be non-functional (and/or dominant-negative); when a translational frameshift occurs at the A_9_ sequence, less than 1/10 of all nascent *lac* repressor sub-units will be non-functional (and/or dominant-negative).

To gain experimental evidence that transcription error and not translational frameshifting promotes stochastic switching in the A_9_ *lacI* allele, we introduced the A_9_ and A_5_GA_3_ *lacI* alleles into a Δ*greAB* strain that contains deletions of the *greA* and *greB* genes that encode auxiliary fidelity factors that facilitate the proofreading of misincorporations that arise in nascent mRNAs during transcription [37]–[39]. We reasoned that if the switching is due to transcription slippage, removing transcription fidelity factors GreAB may promote more slippage and increase phenotypic switching. The increase in switch frequency in the presence of an error-prone sequence in the absence of RNA editing function is more than additive: the median switch frequency for the A_9_ allele in the Δ*greAB* background (42.4%) is greater than the median switch frequency for the A_9_ allele (2.46%) and the Δ*greAB* A_5_GA_3_ background (26.7%) combined (Figure 5A,B). When a slippery transcribed sequence is in a sloppy RNA transcription fidelity background, the switching frequency is significantly increased (Mann-Whitney Rank Sum Test, *p*<0.001), suggesting that misalignments due to transcriptional frameshifting are prevented by RNA editing of the Gre factors *via* transcription back-tracking/correction and support the role of transcription slippages in phenotypic change.

Furthermore, phenotypic switching has been associated with a large burst in permease synthesis [17]. An average gene transcript in *E. coli* will produce about 20–40 full-length polypeptides [40], [41]. Therefore, one transcription frameshift will produce 20–40 non-functional *lac* repressor sub-units (*lac* repressor is a tetramer), while one translational frameshift event will produce only one non-functional *lac* repressor sub-unit along with 20–40 functional *lac* repressor sub-units; a ratio of at least 20∶1 of functional over non-functional *lac* repressor sub-units will be the result. Therefore, the effects of a transcription error will be amplified nonlinearly over the original stochastic event generating tens of aberrant *lac* repressor polypeptides allowing a large burst of permease that is required for phenotypic change [17].

To measure the effect of translation frameshifting on *lac* operon induction, we created merodiploids that provide a 10-fold excess of wild-type transcripts over ±1 frameshift transcripts derived from the *lacI* A_8_ and A_10_ alleles we constructed on the chromosome (Table S2). We introduced an F′ factor with a wild-type *lacI* gene under control of the *I^q^* up-promoter mutation, producing 10-fold more *lacI* transcription [42], into *recA* derivatives of our A_8_ and A_10_ frameshifted *lacI* strains (and into *recA* derivatives of our A_9_ and A_5_GA_3_ in-frame *lacI* strains; see Table S2). Therefore, the ratio of wild-type transcript over frameshifted transcript, at the level of transcription (10∶1), will be a very conservative approximation of the situation that would arise if during the translation of one A_9_ transcript, one translational frameshift event would occur since a ratio of at least 20∶1 of wild-type repressor sub-units over frameshifted sub-units would result. As shown in Figure 5C,D,E, a translation error on a pristine mRNA producing just one aberrant polypeptide amongst many wild-type polypeptides has no large effect on *lac* operon induction, demonstrating that more than one frameshifted repressor sub-unit is required to promote phenotypic switching.

Finally, efficient translation frameshifting is dependant on specific downstream sequence elements in *E. coli* [27] that are not obvious in our A_9_ *lacI* construct.

All together, these results show that insertion of a known transcription slippage sequence in the *lacI* transcript increases phenotypic switching in the bistable *lac* system due to transcription error in the mRNA and accumulation of a frameshifted and non-functional *lac* repressor leading to a transcription burst of *lac* permease.

### Stochastic Errors in Information Transfer Have Heritable Phenotypic Consequences

**Figure 6:**
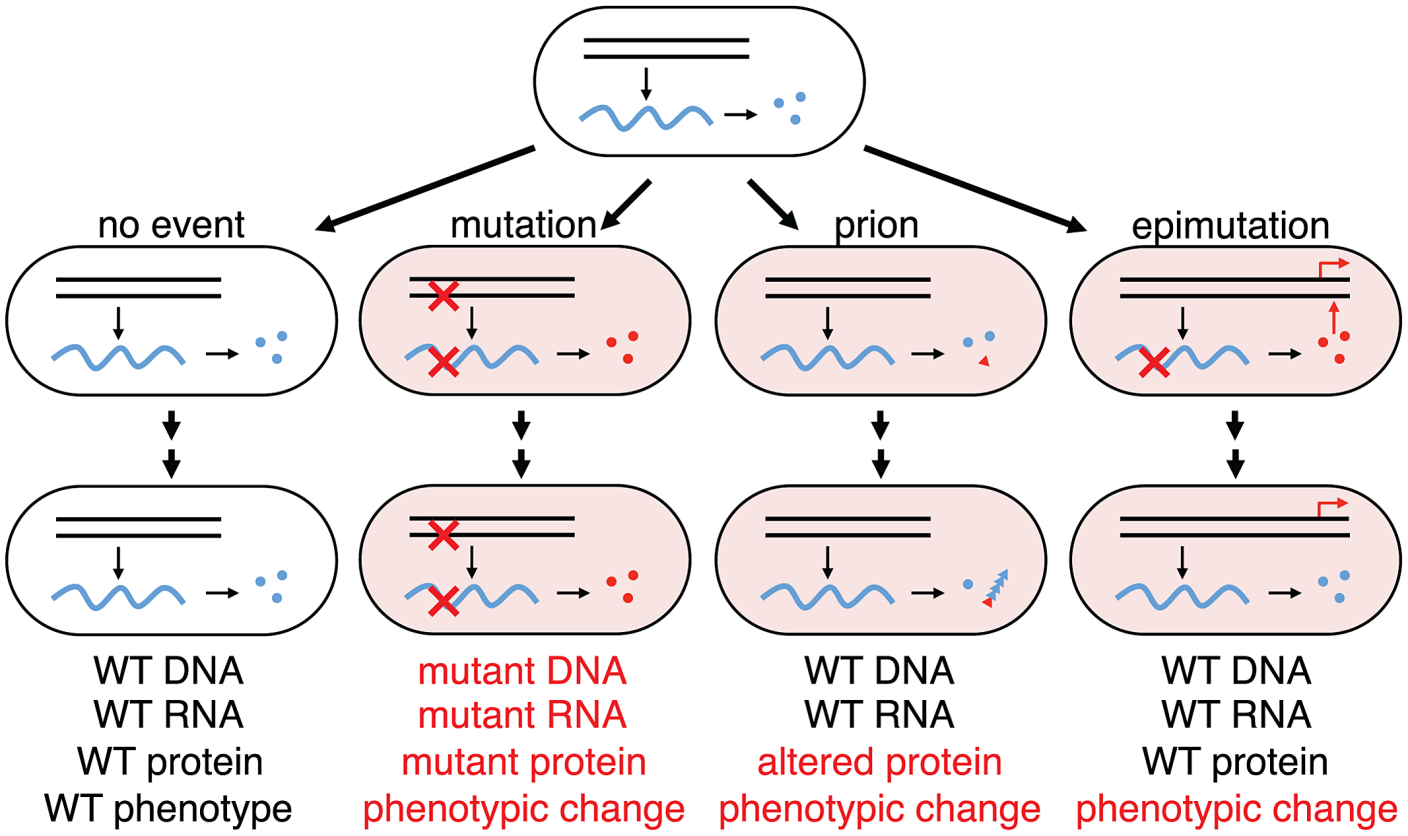
Phenotypic consequences from errors in information transfer in a cellular lineage. Wild-type genes (black parallel lines) make wild-type transcripts (blue wavy lines) make wild-type functional proteins (blue circles); mutant genes make mutant transcripts (red crosses) make mutant proteins (red circles); protein mis-folding can trigger phenotypic change by changing protein conformation to the prion state (red triangle) that can self-perpetuate by templating the aberrant conformation with nascent native proteins (blue triangles). From wild-type genes can also come altered mRNA (epimutation) making altered proteins that can perturb transcriptional networks in a nonlinear manner generating a heritable phenotypic change (red arrows) from a transient stochastic error in information transfer. In this case no trace of the error will remain in the lineage after the phenotypic change as indicated: while change through mutation will retain evidence of the original stochastic error in the progeny cell (mutant DNA, mutant RNA and mutant protein), change through epimutation will retain no evidence of the original stochastic error in the progeny cell (WT DNA, WT RNA and WT protein). Errors in DNA and RNA synthesis occur at rates of, very roughly, 10^−9^ and 10^−5^ errors per residue, respectively [48]; yeast cells in the non-prion [*psi* ^−^] state spontaneously switch [43] to [*PSI* ^+^] at a frequency of 10^−6^; the great majority of cells will not have sustained any errors in information transfer.

DNA makes RNA makes protein; until now, errors in making two of the three elements in information transfer, DNA replication and protein folding, have been shown to modify cellular inheritance through mutation or prion conformational change [43] (Figure 6). Our results show that acute errors in mRNA, the transient element in information transfer, can also effect heritable change when they affect transcription factors involved in bistable gene networks. Transcription errors have been shown to have phenotypic consequences for the cell, but in the cases reported so far, chronic transcription errors can provide partial function or ‘leakiness’ giving an altered phenotype from a mutant or wild type gene [29]–[31], [44]–[46]. For example, a −1 frameshift mutation in the apolipoprotein B gene in a polyA run caused hypobetalipoproteinemia and transcription slippage at the polyA track restores the reading frame by insertion of an additional A and ameliorates the disease [29], [30]. In Alzheimer's patients, it was found that −2 frameshifts accumulated in amyloid precursor and ubiquitin B transcripts over time, which are thought to be important in nonfamilial early- and late-onset forms of Alzheimer's disease [47]. In contrast to these examples of chronic transcriptional errors producing a partial phenotype, we show that one acute transcription error on a poorly transcribed mRNA may promote heritable phenotypic change due to a change of connectivity in a transcription network. Although epimutation, a heritable change in gene expression that does not affect the actual base pair sequence of DNA, is usually associated with methylation patterns and epigenetic silencing of gene expression, the heritable stochastic switching due to transient transcription error we observe here, may also be included as epimutation.

RNA transcription errors are inevitable, ubiquitous and frequent (about 10,000 times more frequent than DNA replication errors) [48] and bistable gene networks sensitive to stochastic fluctuation in protein level are found in bacteria [17], yeast [49], fly [50], *Xenopus* [13], mammalian stem cells [51] and viruses such as HIV and lambda [52]. Furthermore, recent studies in *E. coli* [53], yeast [54] and human cells [55], [56] have shown that a majority of proteins involved in transcription networks are usually present at low abundance so it is easy to imagine that errors in one low abundant mRNA can have a drastic reduction in the protein concentration and trigger epigenetic change. So far, permanent activation or removal of transcription factors by genetic manipulation has been shown to promote a stable change of phenotype in a process known as transdifferentiation [57] and we speculate that transient disappearance of a transcription factor involved in a bistable switch may have the same phenotypic effect. Although here we focus on a slippery A_9_ sequence in the *lacI* gene, any kind of transcription error along the *lacI* transcript that would generate a non-functional *lac* repressor, may also initiate a stochastic switching event. Indeed, any transcribed DNA sequence that is problematic for RNA polymerase, due to a mono-, di-, tri- or some higher order nucleotide repeat or to hairpin or other secondary structures would produce a variety of mRNA species from the same gene. Therefore, transcription errors, epimutation, might be a general mechanism to create epigenetic heterogeneity in a clonal cell population and should be considered as one of the origins of phenotypic change that could lead to altered or aberrant cell behavior, impacting human health, including cancer if the dysregulation of key genetic bistable networks are altered by errors in transcription [58].

## Materials and Methods

### Bacterial Strains

All strains used in this study are derived from the wild-type sequenced *E. coli* MG1655 strain (Table S2). Manipulation of the MG1655 genome was accomplished by standard methodologies [33], [59]. To monitor the proportion of cells that are ON or OFF for *lac* operon expression, we have replaced the *lacA* gene in the wild-type *E. coli* MG1655 chromosome with a *gfp* cassette, so that when the *lacZYA::gfp* transcript is expressed, β-galactosidase, galactoside permease, and green fluorescent protein are produced from the *lacZ*, *lacY*, and *gfp* genes, respectively.

### Growth Conditions and Media

To demonstrate hysteresis and bistability in *lac* operon expression in single cells, a bacterial culture grown in minimal A salts [33] plus MgSO_4_ (1 mM) with succinate (0.2%), was diluted 1∶5 in fresh medium with (ON culture) or without 1 mM TMG (OFF culture) and shaken at 37°C for 7 h. After this induction period, the two cultures were individually diluted and ∼200 cells were seeded to new tubes containing fresh medium that contained varying amounts of TMG, and shaken at 37°C for 42 h (∼28 generations). Flow cytometry was used to determine the percentage of cells that were induced for *lac* operon expression (ON cells).

To determine epigenetic and genetic switch frequencies, a bacterial culture grown in minimal succinate media, was diluted and ∼200 cells were seeded to new tubes containing fresh medium, with (epigenetic assay) or without (genetic assay) a maintenance level of TMG, and shaken at 37°C for 42 h. To determine genetic-mutation frequencies, dilutions of the subcultures were spread on selection plates (minimal A medium supplemented with 75 µg/ml phenyl-β-D-galactoside [Pgal] and 1.5% purified agar) and minimal A glucose (0.2%) plates and incubated for 2–3 days at 37°C. Pgal is a substrate of β-galactosidase and can act as a carbon source but does not induce *lac* operon expression, therefore only cells constitutively expressing β-galactosidase (*lacI^−^*) can form colonies [33].

### Flow Cytometry

To determine the percentage of cells that were induced for *lac* operon expression (ON cells), 10 µl of culture was diluted into 300 µl filtered minimal A salts plus MgSO_4_ (1 mM) and subjected to flow cytometry analysis with GFP fluorescence measured in a BD FACSCanto II Flow Cytometer (Becton, Dickinson and Company, USA) with Diva acquisition software (Becton Dickinson) and FloJo analysis software (Tree Star, Inc. USA). To monitor fluorescent cells in a culture we used a narrow gating for forward and side scattering so that the most represented cell population was evaluated (Figure S1). For each independent culture 10,000 cells were interrogated.

### Microscopy and Microfluidics

To follow the growth of single cells into microcolonies we used the CellASIC ONIX Microfluidic Platform (Millipore) including microfluidic perfusion system, microfluidic flow chamber for bacteria (BO4A plates) and FG software. Time-lapse microscopy was performed using a Zeiss HAL100 inverted fluorescence microscope. Fields were acquired at 100× magnification with an EM-CCD camera (Hammamatsu). Bright field and fluorescence (EGFP cube = Chroma, #41017; X-Cite120 fluorescence illuminator [EXFO Photonic Solutions]) images were acquired and image analysis was performed using AxioVision Rel. 4.6 (Zeiss). To maintain a constant 37°C environment throughout the experiment, the microscope was housed in an incubation system consisting of Incubator XL-S1 (PeCon) controlled by TempModule S and Heating Unit XL S (Zeiss).

## Supporting Information

Figure S1 Gating used in flow cytometry analysis. (A) A forward (FSC) and side scatter (SSC) plot of 10^4^ *lac* operon ON cells. We typically use a flow rate of 2,000 to 5,000 events per second. The gating we use is outlined in black; fully 97% of all events fall within this gating. (B) A FSC and SSC scatter plot of 367 non-cellular events (flow cytometry was performed on filter-sterilized buffer); such events occur typically at 30–50 events per second. When gated as in (A), 29% of all this non-cellular population fall within the gated area. (C) When the 367 non-cellular un-gated events are superimposed over the 10,000 cellular un-gated events, it is apparent that the two populations share a common FSC and SSC space. We superimpose these two populations to provide an estimation of how many false-cellular events to consider in our flow cytometry analyses. Since the number of events we observe per second during analysis of cell populations is about 50–100 times greater than we observe during buffer interrogation, we may therefore reasonably expect that a few percent of our experimental cellular population is actually non-cellular events. (D) When the two un-gated populations are now plotted with SSC against GFP fluorescence, it becomes apparent that the non-cellular events frequently fall in the OFF cellular space, and very infrequently in the ON cellular space. Of the 10,00 cellular events, fully 96% are considered ON; of the non-cellular events fully 1.6% are considered ON (6 events). (E) The gated non-cellular population is now superimposed over the gated cellular population. (F) When the two gated populations are now plotted with SSC versus GFP fluorescence, a more accurate estimation is achieved concerning the number and character of non-cellular events in the experimental population. Therefore, a few percent of the considered experimental population will be non-cellular events (106 non-cellular events in this instance), but only one or two non-cellular events will be considered positive with respect to *lac* operon induction (false-positives in our analysis). With these results we can conclude that for any of our samples, while a few percent of the total from flow cytometry will be false negative with respect to *lac* operon induction, only a few 0.1 percent of the experimental population will be false-positive with respect to *lac* operon induction. Therefore, we have confidence in our experimental procedure, and the results obtained with this procedure, since we are monitoring the *lac* operon OFF to ON stochastic epigenetic switch frequency.
(PDF)

Figure S2 Hysteresis and bistability in the *lac* operon in wild-type and Δ*greAB* cells. (A) Representative flow cytometry GFP fluorescence histogram series of wild-type and Δ*greAB* cells that were originally ON (green histograms) or OFF (red histograms) were sub-cultured and grown in media containing various concentrations of TMG indicated on the vertical axis. Below a concentration of 4 µM TMG and above a concentration of 15 µM TMG, the previous history of the cells (be they originally ON or OFF) is immaterial; between these TMG concentrations the system exhibits hysteresis. The shaded area highlights the maintenance concentration [8], [18] of 6 µM TMG for these strains with the wild-type *lacI* gene (that concentration at which an ON population remains ON, while an OFF population remains OFF; however, an OFF cell has the possibility of switching ON). Each distribution corresponds to GFP fluorescence as measured by flow cytometry of 10^4^ cells of the population grown in the TMG concentration indicated on the vertical axis. (B) Cells that were originally ON or OFF were sub-cultured and grown in media containing various concentrations of TMG. The shaded region shows the maintenance concentration of TMG. Each value is the average ± SD from 5 to 15 independent cultures. (C) Stochastic switching in the *lac* bistable gene network is increased when fidelity of transcription is decreased. When OFF cells are grown in the presence of maintenance concentration of TMG, the absence of GreA and GreB (blue histograms) increases the proportion of ON cells with respect to wild-type cell levels (red histograms). Each blue and red line represents an independent histogram (20 independent cell populations are shown) representing the interrogation of 10^4^ cells; wild-type histograms are super-imposed over Δ*greAB* histograms. The increase in stochastic switch frequency is 38-fold over wild-type level [18]. (D) Absence of GreAB increases stochastic switching in the *lac* operon system. Each value is the median epigenetic-switch frequency from 27 to 30 independent cultures of each strain; the 5% and 95% confidence interval values are included. The mean for the Δ*greA* Δ*greB* strain is significantly different from the wild-type value (Mann-Whitney Rank Sum Test, *p*<0.001) [18].
(PDF)

Figure S3 Creation of a *lacIZYA* operon fusion to assess the levels of gene expression from the *lacI* gene promoter. An operon fusion was created by first inserting a kanamycin cassette from pKD4 (Table S3) into the intervening region between *lacI* and *lacZ* and then, *via* a flippase reaction, removing most of *lac* operator *O_3_*, the complete *lac* promoter and *lac* operator *O_1_*. Therefore, the *lacIZYA* fusion transcript is under the expression of the weakly constitutive *lacI* promoter with no interference from any *lac* repressor binding (*lac* repressor does not negatively regulate *lac* expression through *O_2_* alone) [60]. The complete sequence of the intervening region from the TGA stop codon of the *lacI* gene to the ATG start codon of the *lacZ* gene is shown before and after the fusion was created. Black boxes denote *lac* operator sequences, the green box denotes the *lac* promoter and the purple box denotes the *FRT* sequence left after the kanamycin resistance cassette was flipped out. The kanamycin cassette was amplified using oligos OC365 and OC366 (Table S4) and pKD4 as a template; the homology of the oligos allowed this cassette to be recombined between the STOP codon of *lacI* and the START codon of *lacZ*. The sequences of all constructs were analyzed.
(PDF)

Figure S4 Recombineering an error-prone A_9_ sequence and the broken run A_5_GA_3_ sequence into *lacI* on the *E. coli* chromosome creating a functional *lac* repressor. (A) The approach is outlined; the sequences of the OC primers indicated are found in Table S4; the genotypes of the CH strains are found in Table S2. The phenotype of the original, intermediate and final construct is indicated on the right (Lac, ability to utilize lactose as a carbon source; Cm, sensitivity and resistance to the antibiotic chloramphenicol); R in the altered sequence indicates a purine residue. The entire *lacI* gene, after the initial GTG start codon, along with the first 5 codons of *lacZ*, was replaced with the chloramphenicol resistance gene from pKD3. The A_9_ and A_5_GA_3_ *lacI* alleles were then recombined into the chromosome replacing the *cat* gene and restoring the *lacZ* gene and *lac* operon function. Additionally, we also created A_8_ and A_10_ *lacI* alleles (not shown) at this same site creating frameshifted *lacI* open reading frames with oligos OC 359 and 360, respectively, each with OC 464, to restore *lacZ* with an altered *lacI* allele. The sequences of all constructs were analyzed. (B) N-terminal Lys-Lys appended *lac* repressors are functional. Induced and uninduced populations of wild-type *lacI* cells, and *lacI* A_9_ and A_5_GA_3_ cells, were grown in minimal A salts plus glucose and β-galactosidase levels were determined by the method of Miller [33]; the average ± SD for three independent cultures is shown. This result is entirely consistent with the flow cytometry results presented in Figure 1B. (C) The *lacI* N-terminal Lys-Lys appendage creates an increased tight-binding *lac* repressor. While being a functional *lac* repressor in all aspects, the A_9_/A_5_GA_3_ altered *lac* repressor requires an increased TMG concentration to achieve maintenance (9 µM versus 6 µM for the native *lac* repressor, indicated by the shaded regions); see Table S1. We suggest that the Lys-Lys addition creates a tighter binding *lac* repressor. Most amino acid substitutions at positions 2, 3 or 4 of the *lac* repressor result in a wild-type phenotype or in a tight-binding phenotype [62]. Moreover, the amino terminus of the λ repressor has the sequence NH_2_-Ser-Thr-Lys-Lys-Lys-Pro- which is similar to the sequence of the A_9_/A_5_GA_3_ altered *lac* repressor, namely NH_2_-Met-Lys-Lys-Lys-Pro-; it is this Lys-Lys-Lys amino acid sequence that forms the arm of the λ repressor that wraps around the DNA adding additional protein∶DNA contacts to augment the usual helix-turn-helix DNA-binding motif [63]. Essentially, *via* the Lys-Lys addition generating a Lys-Lys-Lys run, we have tacked on a flexible DNA-binding segment to the *lac* repressor and this is reflected in the increased maintenance concentration observed for these cells. Each value is the average ± SD from 5 to 20 independent cultures.
(PDF)

Figure S5 The error-prone A_9_ run in the *lacI* transcript increases stochastic phenotypic switching. (A) Uninduced (OFF) A_5_GA_3_ *lacI* cells (red histograms) and A_9_ *lacI* cells (blue histograms) were diluted and grown in media containing 10 µM TMG. After 42 h growth, flow cytometry was performed to determine the frequency of epigenetically ON cells in 20 independent cultures of each strain; the histograms from the A_9_ *lacI* cultures are superimposed over the histograms from A_5_GA_3_ *lacI* cultures; each histogram represents the interrogation of 10^4^ cells. (B) The Y axis scale is changed from a maximum of 250 cells to 60 cells to allow a close examination of the resulting histograms, clearly showing that the A_9_ run in the *lacI* transcript increases stochastic phenotypic switching. (C) Each value is the median epigenetic-switch frequency from 20 independent cultures of each strain; the 5% and 95% confidence interval values are included. The mean for the A_9_ *lacI* strain is significantly different from the A_5_GA_3_ *lacI* value (Mann-Whitney Rank Sum Test, *p*<0.001).
(PDF)

Figure S6 The *lacIZYA* operon fusion: The nature of the *lacI* sequence does not affect downstream *lacZYA* operon expression (*i.e.* frameshift events at the A_9_ run are not polar). A novel *lacI^+^-ZYA* operon fusion, and altered *lacI-ZYA* fusion derivatives, was created to determine if read-through transcription that initiates at the *lacI* promoter and continues into the *lac* operon was affected by transcriptional frameshift events at the 5′ end of the *lacI* mRNA. Details of the fusion construction are shown in Figure S3. The native *lacI* transcript is not terminated by a transcriptional terminator sequence, but instead transcription is terminated when the RNA polymerase encounters *lac* repressor bound to the *lac* operator [64], [65]; read-through transcription is thought to be responsible for the basal levels of *lac* operon activities in uninduced cells [64], [65], and therefore transcriptional events that affect *lacI* transcript stability would also affect the basal levels of *lac* functions, and perturb the normal system. In the fusion strains, the *lac* promoter, all of *lac* operator *O*_1_ and half of *O*_3_ have been replaced with an FRT sequence, and therefore transcription that initiates at the *lacI* promoter will continue through the *lacZYA* operon creating a novel operon, *lacIZYA*, and a transcript encoding the *lac* repressor, β-galactosidase, permease and transacetylase. Operator *O*_2_, in the absence of functional *O*_1_ and *O*_3_ operators, does not possess significant operator function and *O*_2_ alone does not exert detectable repression [60]. The altered *lacI* alleles are modified immediately after the *lacI* GUG initiation codon and include the addition of six, seven and five A residues (to create monotonic runs of A_9_, A_10_, A_8_, respectively). The in-frame A_9_ allele adds two additional Lys residues to the repressor; the in-frame A_5_GA_3_ allele also adds an additional two Lys residues but the A_9_ run is interrupted. The out-of-frame A_8_ allele would cause translation to terminate at codon 4/5; the out-of-frame A_10_ allele would cause translation to terminate at codon 83/84. All the *lacI-ZYA* operon fusions exhibit similar constitutive β-galactosidase activity levels demonstrating that ±1 frameshift events in the A_9_ *lacI* allele, generating A_8_ and A_10_ runs, are not polar, do not affect the fusion mRNA stability and do not affect expression of downstream *lacZYA* genes in the single transcript. Cells were grown in minimal A salts plus glucose and β-galactosidase levels were determined by the method of Miller [33]; the average ± SD for three independent cultures is shown. Constitutive transcription in all the *lacIZYA* operon fusion strains also provides enough Lac function for colonies to grow slowly on lactose agar plates, and to appear blue on glucose 5-bromo-4-chloro-3-indolyl-β-D-galactoside (Xgal) indicator plates, with or without inducer (data not shown). Therefore, these operon fusions demonstrate our system is competent for the study of transcriptional frameshift events.
(PDF)

Movie S1 Stochastic switching of one cell in a clonal population in the same environment. The growth of a single *lacI* A_9_ OFF cell in TMG maintenance media into a microcolony was followed in a microfluidics flow chamber by time-lapse microscopy. Each frame was, on average, taken about 60 minutes apart (some frames were lost due to loss of focus). The bright field frames are on the left, the GFP fluorescence frames are on the right and begin after the microcolony has become well established. The cell outlined in yellow is the cell that will change phenotype from OFF to ON, and the descendants of that cell will also manifest the ON phenotype. The cell outlined in red is the direct relative of the cell that switches ON, but that cell, and all the descendants of that cell remain OFF. All the GFP fluorescence frames, until the last frame, have 100 ms exposure times and the resulting images were over-exposed using the ColorSync Utility to observe the faint fluorescence signal. The last GFP fluorescence frame had a 3000 ms exposure time. The movie shows that an OFF cell growing in TMG maintenance media will be OFF, but has the possibility of switching ON, and once switched ON will initiate a cell lineage of ON cells, even though other surrounding cells in the same microcolony will be OFF.
(MOV)

Table S1 Maintenance of ON phenotype of cells grown in 9 µM TMG. To demonstrate maintenance of *lac* operon expression in single cells, an overnight bacterial culture of the strain of interest carrying the *lacZYA::gfp* construct, inoculated from a single colony and grown in minimal succinate media, was diluted 1∶5 in fresh medium with 1 mM TMG (ON culture) and shaken at 37°C for 7 h. After this induction period, the cultures were individually diluted and ∼200 cells were seeded to new tubes containing fresh medium that contained 9 µM TMG, and shaken at 37°C for 42 h. To determine the percentage of cells that remained ON for *lac* operon expression, 10 µl of culture was diluted into 300 µl filtered minimal A salts plus MgSO_4_ (1 mM) and subjected to flow cytometry analysis with a BD FACSCanto II Flow Cytometer (Becton, Dickinson and Company, USA). At this TMG concentration, all the strains exhibit maintenance (over 90% of the original ON cells and their descendants remain ON after prolonged growth at 9 µM TMG), and can be considered as samples of the same population and therefore directly compared, since the differences between the populations are not significant (Kruskal-Wallis One Way Analysis of Variance on Ranks, *p* = 0.10). Each value is the median from 5–25 independent cultures; the 5% and 95% confidence interval values are indicated.
(PDF)

Table S2 Bacterial strains.
(PDF)

Table S3 Plasmids.
(PDF)

Table S4 Oligos.
(PDF)

Text S1 Considering *lacI* ^+^→*lacI* ^−^ forward mutation frequency analysis.
(DOCX)

Text S2 References for supporting information.
(DOCX)
