# Supplementary figures and images for: Heritable Change Caused by Transient Transcription Errors
Source: PLoS Genet. 2013 Jun 27;9(6):e1003595. doi: 10.1371/journal.pgen.1003595 (PMC3694819; doi:10.1371/journal.pgen.1003595)

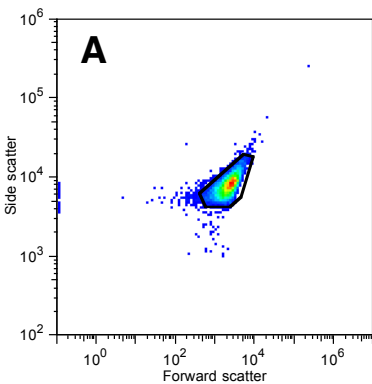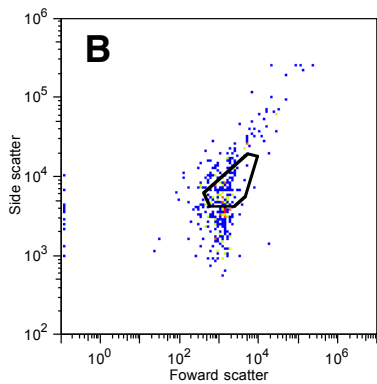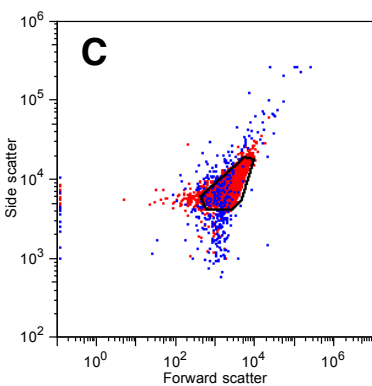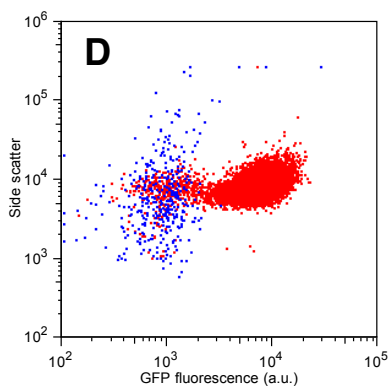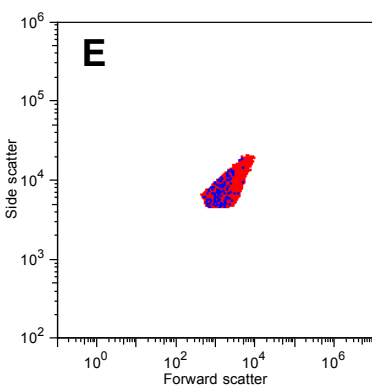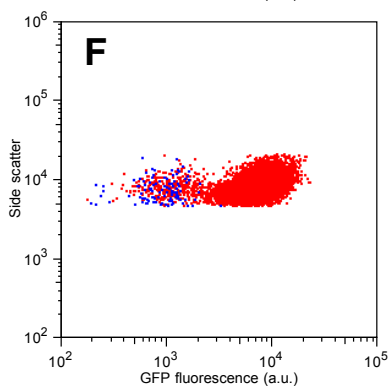

Supplement: Figure S1 — Gating used in flow cytometry analysis. (A) A forward (FSC) and side scatter (SSC) plot of 104 lac operon ON cells. We typically use a flow rate of 2,000 to 5,000 events per second. The gating we use is outlined in black; fully 97% of all events fall within this gating. (B) A FSC and SSC scatter plot of 367 non-cellular events (flow cytometry was performed on filter-sterilized buffer); such events occur typically at 30–50 events per second. When gated as in (A), 29% of all this non-cellular population fall within the gated area. (C) When the 367 non-cellular un-gated events are superimposed over the 10,000 cellular un-gated events, it is apparent that the two populations share a common FSC and SSC space. We superimpose these two populations to provide an estimation of how many false-cellular events to consider in our flow cytometry analyses. Since the number of events we observe per second during analysis of cell populations is about 50–100 times greater than we observe during buffer interrogation, we may therefore reasonably expect that a few percent of our experimental cellular population is actually non-cellular events. (D) When the two un-gated populations are now plotted with SSC against GFP fluorescence, it becomes apparent that the non-cellular events frequently fall in the OFF cellular space, and very infrequently in the ON cellular space. Of the 10,00 cellular events, fully 96% are considered ON; of the non-cellular events fully 1.6% are considered ON (6 events). (E) The gated non-cellular population is now superimposed over the gated cellular population. (F) When the two gated populations are now plotted with SSC versus GFP fluorescence, a more accurate estimation is achieved concerning the number and character of non-cellular events in the experimental population. Therefore, a few percent of the considered experimental population will be non-cellular events (106 non-cellular events in this instance), but only one or two non-cellular events will be conside [file pgen.1003595.s001.pdf]

**A**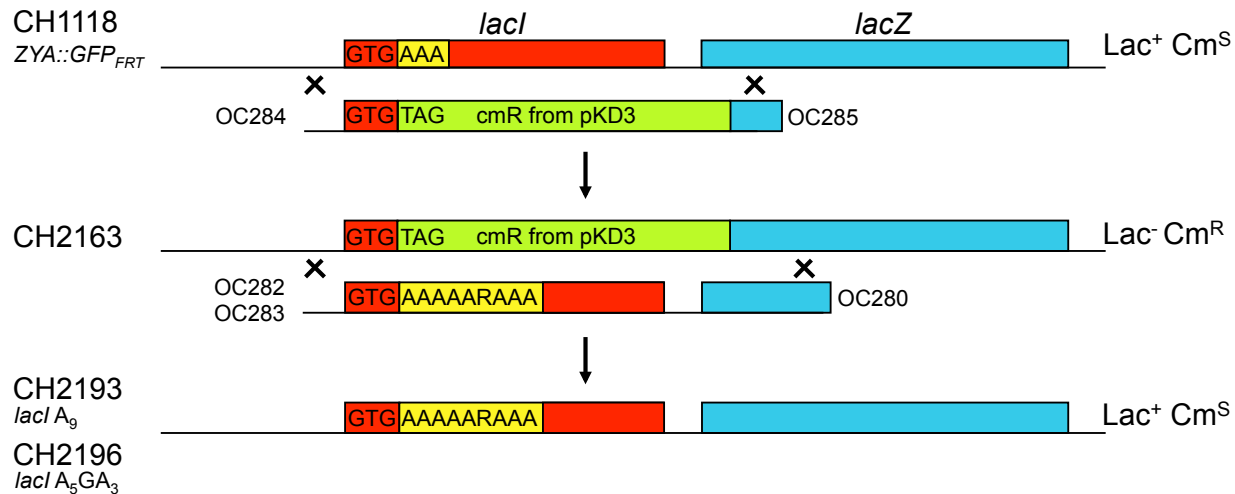**B**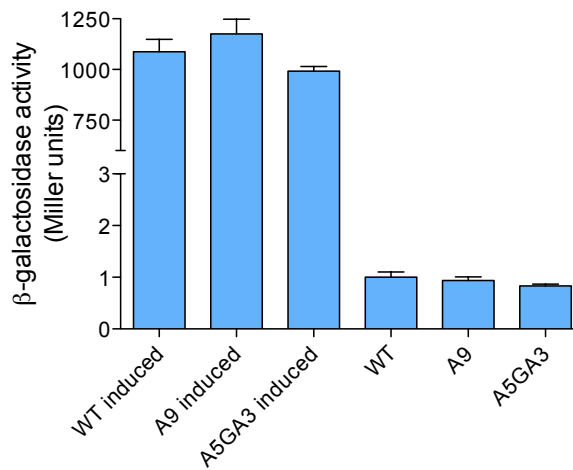**C**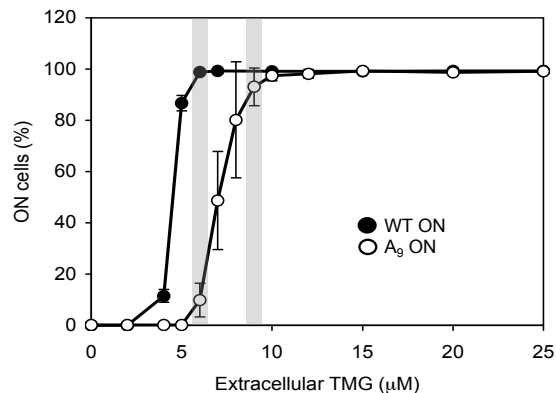

Supplement: Figure S4 — Recombineering an error-prone A9 sequence and the broken run A5GA3 sequence into lacI on the E. coli chromosome creating a functional lac repressor. (A) The approach is outlined; the sequences of the OC primers indicated are found in Table S4; the genotypes of the CH strains are found in Table S2. The phenotype of the original, intermediate and final construct is indicated on the right (Lac, ability to utilize lactose as a carbon source; Cm, sensitivity and resistance to the antibiotic chloramphenicol); R in the altered sequence indicates a purine residue. The entire lacI gene, after the initial GTG start codon, along with the first 5 codons of lacZ, was replaced with the chloramphenicol resistance gene from pKD3. The A9 and A5GA3 lacI alleles were then recombined into the chromosome replacing the cat gene and restoring the lacZ gene and lac operon function. Additionally, we also created A8 and A10 lacI alleles (not shown) at this same site creating frameshifted lacI open reading frames with oligos OC 359 and 360, respectively, each with OC 464, to restore lacZ with an altered lacI allele. The sequences of all constructs were analyzed. (B) N-terminal Lys-Lys appended lac repressors are functional. Induced and uninduced populations of wild-type lacI cells, and lacI A9 and A5GA3 cells, were grown in minimal A salts plus glucose and β-galactosidase levels were determined by the method of Miller [33]; the average ± SD for three independent cultures is shown. This result is entirely consistent with the flow cytometry results presented in Figure 1B. (C) The lacI N-terminal Lys-Lys appendage creates an increased tight-binding lac repressor. While being a functional lac repressor in all aspects, the A9/A5GA3 altered lac repressor requires an increased TMG concentration to achieve maintenance (9 µM versus 6 µM for the native lac repressor, indicated by the shaded regions); see Table S1. We suggest that the Lys-Lys addition creates a tighter binding lac repressor. Most amino [file pgen.1003595.s004.pdf]

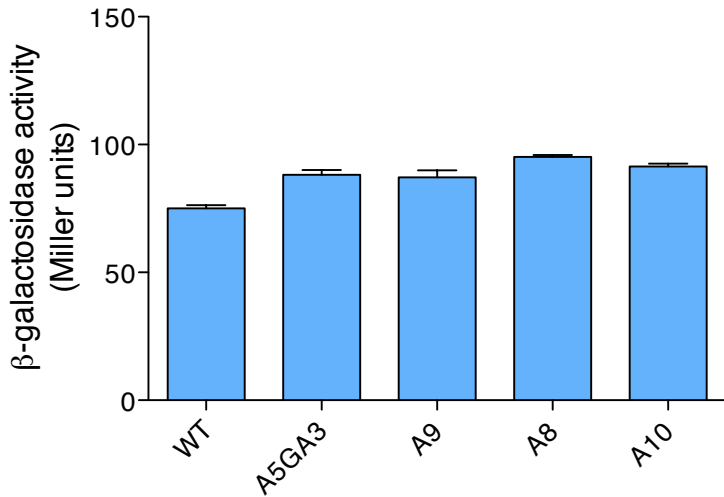

Supplement: Figure S6 — The lacIZYA operon fusion: The nature of the lacI sequence does not affect downstream lacZYA operon expression (i.e. frameshift events at the A9 run are not polar). A novel lacI+-ZYA operon fusion, and altered lacI-ZYA fusion derivatives, was created to determine if read-through transcription that initiates at the lacI promoter and continues into the lac operon was affected by transcriptional frameshift events at the 5′ end of the lacI mRNA. Details of the fusion construction are shown in Figure S3. The native lacI transcript is not terminated by a transcriptional terminator sequence, but instead transcription is terminated when the RNA polymerase encounters lac repressor bound to the lac operator [64], [65]; read-through transcription is thought to be responsible for the basal levels of lac operon activities in uninduced cells [64], [65], and therefore transcriptional events that affect lacI transcript stability would also affect the basal levels of lac functions, and perturb the normal system. In the fusion strains, the lac promoter, all of lac operator O 1 and half of O 3 have been replaced with an FRT sequence, and therefore transcription that initiates at the lacI promoter will continue through the lacZYA operon creating a novel operon, lacIZYA, and a transcript encoding the lac repressor, β-galactosidase, permease and transacetylase. Operator O 2, in the absence of functional O 1 and O 3 operators, does not possess significant operator function and O 2 alone does not exert detectable repression [60]. The altered lacI alleles are modified immediately after the lacI GUG initiation codon and include the addition of six, seven and five A residues (to create monotonic runs of A9, A10, A8, respectively). The in-frame A9 allele adds two additional Lys residues to the repressor; the in-frame A5GA3 allele also adds an additional two Lys residues but the A9 run is interrupted. The out-of-frame A8 allele would cause translation to terminate at codon 4/5; the out-of-frame [file pgen.1003595.s006.pdf]
