# Supplementary material for: Heritable Change Caused by Transient Transcription Errors
Source: PLoS Genet. 2013 Jun 27;9(6):e1003595. doi: 10.1371/journal.pgen.1003595 (PMC3694819; doi:10.1371/journal.pgen.1003595)
Supplement: Figure S2 — Hysteresis and bistability in the lac operon in wild-type and ΔgreAB cells. (A) Representative flow cytometry GFP fluorescence histogram series of wild-type and ΔgreAB cells that were originally ON (green histograms) or OFF (red histograms) were sub-cultured and grown in media containing various concentrations of TMG indicated on the vertical axis. Below a concentration of 4 µM TMG and above a concentration of 15 µM TMG, the previous history of the cells (be they originally ON or OFF) is immaterial; between these TMG concentrations the system exhibits hysteresis. The shaded area highlights the maintenance concentration [8], [18] of 6 µM TMG for these strains with the wild-type lacI gene (that concentration at which an ON population remains ON, while an OFF population remains OFF; however, an OFF cell has the possibility of switching ON). Each distribution corresponds to GFP fluorescence as measured by flow cytometry of 104 cells of the population grown in the TMG concentration indicated on the vertical axis. (B) Cells that were originally ON or OFF were sub-cultured and grown in media containing various concentrations of TMG. The shaded region shows the maintenance concentration of TMG. Each value is the average ± SD from 5 to 15 independent cultures. (C) Stochastic switching in the lac bistable gene network is increased when fidelity of transcription is decreased. When OFF cells are grown in the presence of maintenance concentration of TMG, the absence of GreA and GreB (blue histograms) increases the proportion of ON cells with respect to wild-type cell levels (red histograms). Each blue and red line represents an independent histogram (20 independent cell populations are shown) representing the interrogation of 104 cells; wild-type histograms are super-imposed over ΔgreAB histograms. The increase in stochastic switch frequency is 38-fold over wild-type level [18]. (D) Absence of GreAB increases stochastic switching in the lac operon system. Each value is the media [file pgen.1003595.s002.pdf]

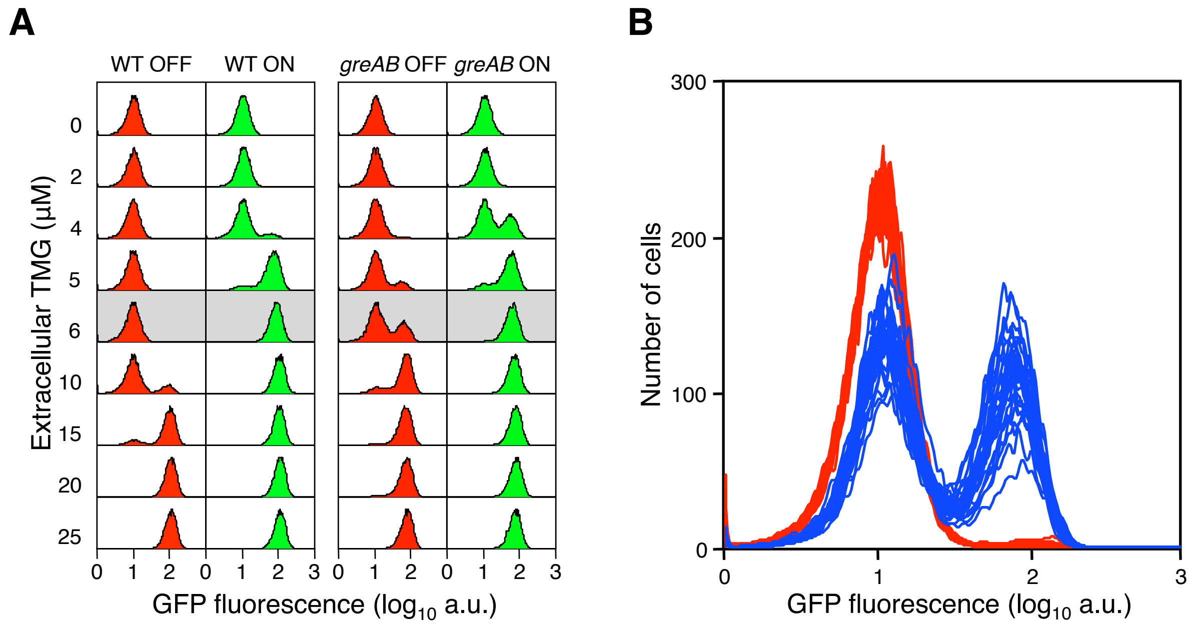

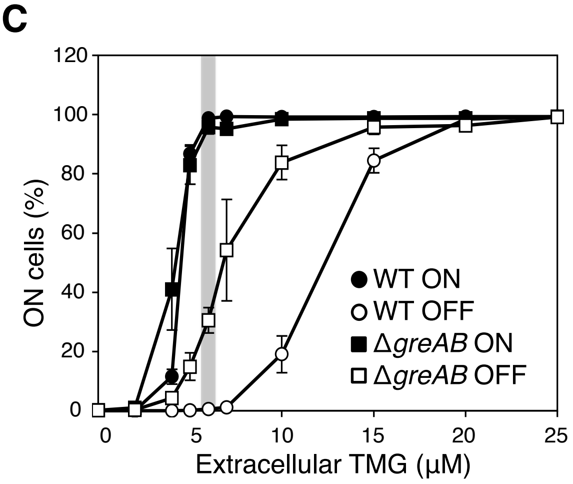

D

Epigenetic switch frequency ($\times 10^{-2}$)

strain	median	5%	95%
WT	1.03	0.46	1.75
Δ*greAB*	41.00	25.26	56.81

p = < 0.001 (Mann-Whitney Rank Sum Test)
38.4-fold increase in switch frequency
27-30 independent cultures each strain
