# Supplementary material for: Heritable Change Caused by Transient Transcription Errors
Source: PLoS Genet. 2013 Jun 27;9(6):e1003595. doi: 10.1371/journal.pgen.1003595 (PMC3694819; doi:10.1371/journal.pgen.1003595)
Supplement: Figure S3 — Creation of a lacIZYA operon fusion to assess the levels of gene expression from the lacI gene promoter. An operon fusion was created by first inserting a kanamycin cassette from pKD4 (Table S3) into the intervening region between lacI and lacZ and then, via a flippase reaction, removing most of lac operator O3, the complete lac promoter and lac operator O1. Therefore, the lacIZYA fusion transcript is under the expression of the weakly constitutive lacI promoter with no interference from any lac repressor binding (lac repressor does not negatively regulate lac expression through O2 alone) [60]. The complete sequence of the intervening region from the TGA stop codon of the lacI gene to the ATG start codon of the lacZ gene is shown before and after the fusion was created. Black boxes denote lac operator sequences, the green box denotes the lac promoter and the purple box denotes the FRT sequence left after the kanamycin resistance cassette was flipped out. The kanamycin cassette was amplified using oligos OC365 and OC366 (Table S4) and pKD4 as a template; the homology of the oligos allowed this cassette to be recombined between the STOP codon of lacI and the START codon of lacZ. The sequences of all constructs were analyzed. (PDF) [file pgen.1003595.s003.pdf]

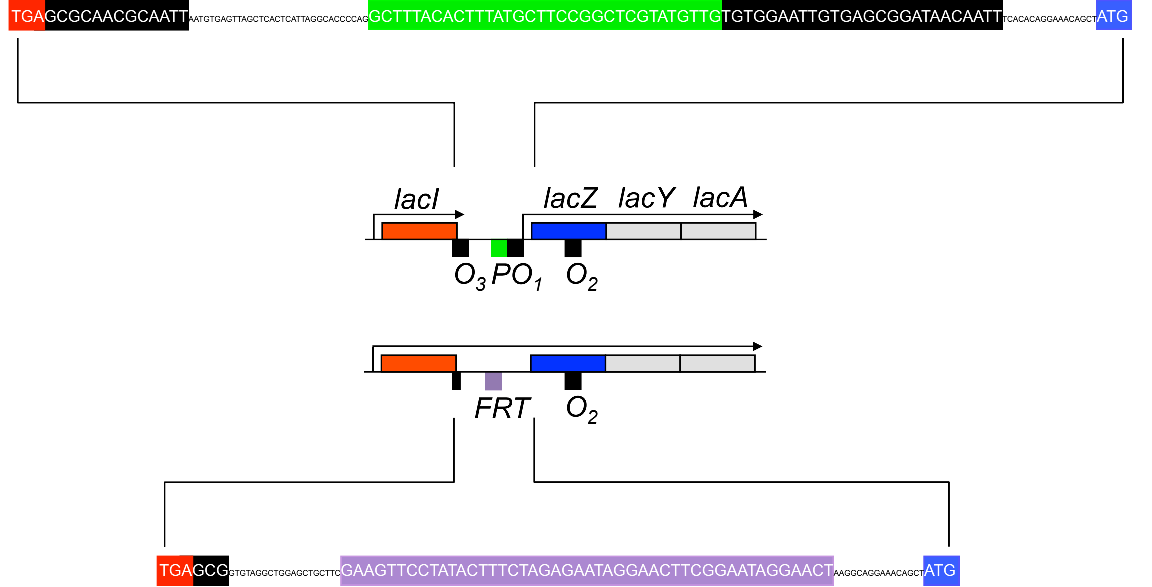

TGAGCGCAACGCAATTAATGTGAGTTAGCTCACTCATTAGGCACCCCAGGCTTTACACTTTATGCTTCCGGCTCGTATGTTGTGTGGAATTGTGAGCGGATAACAATTTCACACAGGAAACAGCTATG
lacI
lacZ lacY lacA
O_3 P O_1 O_2
FRT O_2
TGAGCGGTGTAGGCTGGAGCTGCTTCGAAGTTCCTATACTTTCTAGAGAATAGGAACTTCGGAATAGGAACTAAGGCAGGAAACAGCTATG
