# Supplementary material for: Heritable Change Caused by Transient Transcription Errors
Source: PLoS Genet. 2013 Jun 27;9(6):e1003595. doi: 10.1371/journal.pgen.1003595 (PMC3694819; doi:10.1371/journal.pgen.1003595)
Supplement: Figure S5 — The error-prone A9 run in the lacI transcript increases stochastic phenotypic switching. (A) Uninduced (OFF) A5GA3 lacI cells (red histograms) and A9 lacI cells (blue histograms) were diluted and grown in media containing 10 µM TMG. After 42 h growth, flow cytometry was performed to determine the frequency of epigenetically ON cells in 20 independent cultures of each strain; the histograms from the A9 lacI cultures are superimposed over the histograms from A5GA3 lacI cultures; each histogram represents the interrogation of 104 cells. (B) The Y axis scale is changed from a maximum of 250 cells to 60 cells to allow a close examination of the resulting histograms, clearly showing that the A9 run in the lacI transcript increases stochastic phenotypic switching. (C) Each value is the median epigenetic-switch frequency from 20 independent cultures of each strain; the 5% and 95% confidence interval values are included. The mean for the A9 lacI strain is significantly different from the A5GA3 lacI value (Mann-Whitney Rank Sum Test, p<0.001). (PDF) [file pgen.1003595.s005.pdf]

A B

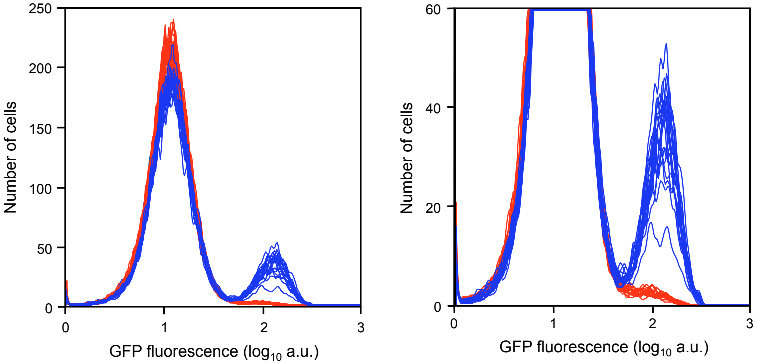

C

Epigenetic switch frequency ($x10^{-2}$)

strain	median	5%	95%
A_9	13.5	8.1	16.6
A_5GA_3	1.1	0.8	1.3

p = < 0.001 (Mann-Whitney Rank Sum Test)
12.0-fold increase in switch frequency
20 independent cultures each strain
