# Supplementary material for: Heritable Change Caused by Transient Transcription Errors
Source: PLoS Genet. 2013 Jun 27;9(6):e1003595. doi: 10.1371/journal.pgen.1003595 (PMC3694819; doi:10.1371/journal.pgen.1003595)
Supplement: Table S1 — Maintenance of ON phenotype of cells grown in 9 µM TMG. To demonstrate maintenance of lac operon expression in single cells, an overnight bacterial culture of the strain of interest carrying the lacZYA::gfp construct, inoculated from a single colony and grown in minimal succinate media, was diluted 1∶5 in fresh medium with 1 mM TMG (ON culture) and shaken at 37°C for 7 h. After this induction period, the cultures were individually diluted and ∼200 cells were seeded to new tubes containing fresh medium that contained 9 µM TMG, and shaken at 37°C for 42 h. To determine the percentage of cells that remained ON for lac operon expression, 10 µl of culture was diluted into 300 µl filtered minimal A salts plus MgSO4 (1 mM) and subjected to flow cytometry analysis with a BD FACSCanto II Flow Cytometer (Becton, Dickinson and Company, USA). At this TMG concentration, all the strains exhibit maintenance (over 90% of the original ON cells and their descendants remain ON after prolonged growth at 9 µM TMG), and can be considered as samples of the same population and therefore directly compared, since the differences between the populations are not significant (Kruskal-Wallis One Way Analysis of Variance on Ranks, p = 0.10). Each value is the median from 5–25 independent cultures; the 5% and 95% confidence interval values are indicated. (PDF) [file pgen.1003595.s008.pdf]

Maintenance of ON phenotype ($x10^{-2}$)			
strain	median	5%	95%
A_9	96.8	76.1	99.3
A_5GA_3	90.6	83.5	98.2
A_9 Δ*greAB*	93.7	91.7	98.9
A_5GA_3 Δ*greAB*	96.1	95.1	97.2
