# Supplementary material for: Heritable Change Caused by Transient Transcription Errors
Source: PLoS Genet. 2013 Jun 27;9(6):e1003595. doi: 10.1371/journal.pgen.1003595 (PMC3694819; doi:10.1371/journal.pgen.1003595)
Supplement: Table S2 — Bacterial strains. (PDF) [file pgen.1003595.s009.pdf]

strain	genotype	reference
**CH30**	MG1655	laboratory stock
**CH4345**	XL1-Blue (Stratagene®)	laboratory stock
**CH458**	*lacZYA*::*gfp-cmR*	[18]
**CH568**	Δ*greA*$_{FRT}$ Δ*greB*$_{FRT}$ *lacZYA*::*gfp-cmR*	[18]
**CH1118**	*lacZYA*::*gfp*$_{FRT}$	[18]
**CH495**	Δ*greA*$_{FRT}$ Δ*greB*$_{FRT}$	[18]
**CH440**	*rpoB* (*rifR*) *lacZ*$_{U118}$ *thr*$^-$ *leu*$^+$ *his*$^-$ *arg*$^-$ *thi*$^-$ *relA*2	[18]
**CH598**	Δ*greA*$_{FRT}$ Δ*greB*$_{FRT}$ *proC*::*Tn5*	CH495 x P1 (CAG39496)
**CH599**	Δ*greA*$_{FRT}$ Δ*greB*$_{FRT}$ *lacZ*$_{U118}$	CH598 x P1 (CH440)
**CH2350**	Δ*greA*$_{FRT}$ Δ*greB*$_{FRT}$ *lacZ*$_{U118}$ *proC*::*Tn5*	CH599 x P1 (CH598)
**CH2163**	Δ*lacI*::*cmR* *lacZYA*::*gfp*$_{FRT}$	CH1118 x OC284-*cmR*-OC285
**CH2193**	A_9*lacI* *lacZYA*::*gfp*$_{FRT}$	CH2163 x OC282-*lacI*-OC464
**CH2196**	A_5GA_3*lacI* *lacZYA*::*gfp*$_{FRT}$	CH2163 x OC283-*lacI*-OC464
**CH2661**	A_8*lacI* *lacZYA*::*gfp*$_{FRT}$	CH2163 x OC359-*lacI*-OC464
**CH2659**	A_{10}*lacI* *lacZYA*::*gfp*$_{FRT}$	CH2163 x OC360-*lacI*-OC464
**CH2620**	A_9*lacI* *lacZYA*::*gfp*$_{FRT}$ pKD46	CH2193 + pKD46
**CH2621**	A_5GA_3*lacI* *lacZYA*::*gfp*$_{FRT}$ pKD46	CH2196 + pKD46
**CH2664**	A_8*lacI* *lacZYA*::*gfp*$_{FRT}$ pKD46	CH2661 + pKD46
**CH2663**	A_{10}*lacI* *lacZYA*::*gfp*$_{FRT}$ pKD46	CH2659 + pKD46
**CH2667**	A_9*lacI-knR-lacZYA*::*gfp*$_{FRT}$	CH2620 x OC365-*knR*-OC366
**CH2704**	A_5GA_3*lacI-knR-lacZYA*::*gfp*$_{FRT}$	CH2621 x OC365-*knR*-OC366
**CH2712**	A_8*lacI-knR-lacZYA*::*gfp*$_{FRT}$	CH2664 x OC365-*knR*-OC366
**CH2668**	A_{10}*lacI-knR-lacZYA*::*gfp*$_{FRT}$	CH2663 x OC365-*knR*-OC366
**CH2671**	A_9*lacI-knR-lacZYA*	CH30 x P1 (CH2667)
**CH2726**	A_5GA_3*lacI-knR-lacZYA*	CH30 x P1 (CH2704)
**CH2724**	A_8*lacI-knR-lacZYA*	CH30 x P1 (CH2712)
**CH2673**	A_{10}*lacI-knR-lacZYA*	CH30 x P1 (CH2668)
**CH2678**	A_9*lacI*$_{FRT}$*lacZYA*	CH2671 flipped
**CH2751**	A_5GA_3*lacI*$_{FRT}$*lacZYA*	CH2726 flipped
**CH2749**	A_8*lacI*$_{FRT}$*lacZYA*	CH2724 flipped
**CH2680**	A_{10}*lacI*$_{FRT}$*lacZYA*	CH2673 flipped
**CH2358**	Δ*greA*$_{FRT}$ Δ*greB*$_{FRT}$ A_9*lacI* *lacZYA*::*gfp*$_{FRT}$	CH2350 x P1 (CH2193)
**CH2360**	Δ*greA*$_{FRT}$ Δ*greB*$_{FRT}$ A_5GA_3*lacI* *lacZYA*::*gfp*$_{FRT}$	CH2350 x P1 (CH2196)
**CH4048**	*recA::knR*	JW2669 [66]
**CH4088**	*recA::knR*; F' *recA*$^+$	CH4048 + F' *recA*$^+$
**CH4346**	A_9*lacI* *lacZYA*::*gfp*$_{FRT}$ *recA::knR*	CH2193 x P1 (CH4088)
**CH4347**	A_5GA_3*lacI* *lacZYA*::*gfp*$_{FRT}$ *recA::knR*	CH2196 x P1 (CH4088)
**CH4348**	A_8*lacI* *lacZYA*::*gfp*$_{FRT}$ *recA::knR*	CH2661 x P1 (CH4088)
**CH4349**	A_{10}*lacI* *lacZYA*::*gfp*$_{FRT}$ *recA::knR*	CH2659 x P1 (CH4088)
**CH4346**	A_9*lacI* *lacZYA*::*gfp*$_{FRT}$ *recA::knR*; F' *tetR* *lacI*^{q}	CH2193 + F' (XL1-Blue)
**CH4347**	A_5GA_3*lacI* *lacZYA*::*gfp*$_{FRT}$ *recA::knR*; F' *tetR* *lacI*^{q}	CH2196 + F' (XL1-Blue)
**CH4348**	A_8*lacI* *lacZYA*::*gfp*$_{FRT}$ *recA::knR*; F' *tetR* *lacI*^{q}	CH2661 + F' (XL1-Blue)
**CH4349**	A_{10}*lacI* *lacZYA*::*gfp*$_{FRT}$ *recA::knR*; F' *tetR* *lacI*^{q}	CH2659 + F' (XL1-Blue)
**CH2677**	*lacI-knR-lacZYA*	CH30 x OC365-*knR*-OC366
**CH2714**	*lacI*$_{FRT}$*lacZYA*	CH2677 flipped
