# Supplementary material for: Heritable Change Caused by Transient Transcription Errors
Source: PLoS Genet. 2013 Jun 27;9(6):e1003595. doi: 10.1371/journal.pgen.1003595 (PMC3694819; doi:10.1371/journal.pgen.1003595)
Supplement: Table S3 — Plasmids. (PDF) [file pgen.1003595.s010.pdf]

| plasmid | construct | reference |
|---|---|---|
| **pKD3** | *cmR* | [59] |
| **pKD4** | *knR* | [59] |
| **pKD46** | red gam | [59] |
| **pCP20** | flippase | [59] |
