# Supplementary material for: Heritable Change Caused by Transient Transcription Errors
Source: PLoS Genet. 2013 Jun 27;9(6):e1003595. doi: 10.1371/journal.pgen.1003595 (PMC3694819; doi:10.1371/journal.pgen.1003595)
Supplement: Table S4 — Oligos. (PDF) [file pgen.1003595.s011.pdf]

oligo	sequence	reference
**OC282**	5’GGTATGGCATGATAGCGCCCGGAAGAGAGTCAATTCAGGGTGGTGAATGTGAAAAAAAAACCAGTAACGTTATACGATG3’	recombineering of A_9*lacI* (left)
**OC283**	5’GGTATGGCATGATAGCGCCCGGAAGAGAGTCAATTCAGGGTGGTGAATGTGAAAAAGAAACCAGTAACGTTATACGATG3’	recombineering of A_5GA_3*lacI* (left)
**OC284**	5’GGTATGGCATGATAGCGCCCGGAAGAGAGTCAATTCAGGGTGGTGAATGTGTAGGCTGGAGCTGCTTC3’	Δ*lacI*-‘*lacZ*::*cmR* recombineering (left)
**OC285**	5’CCAGGGTTTTCCCAGTCACGACGTTGTAAAACGACGGCCAGTGAATCCCGTATGAATATCCTCCTTAG3’	Δ*lacI*-‘*lacZ*::*cmR* recombineering (right)
**OC287**	5’CTATTCTGGTGGCCGGAAG3’	sequencing of *lacI* start (left)
**OC288**	5’GCCGCTTCCACTTTTTCC3’	sequencing of *lacI* start (right)
**OC289**	5’AAATGCTGAATGAGGGCATC3’	sequencing of *lacI*-*lacZYA* fusion (left)
**OC290**	5’GCATTAATGAATCGGCCAAC3’	sequencing of *lacI*-*lacZYA* fusion (right)
**OC359**	5’GTATGGCATGATAGCGCCCGGAAGAGAGTCAATTCAGGGTGGTGAATGTGAAAAAAAACCAGTAACGTTATACGATG3’	recombineering of A_8*lacI* (left)
**OC360**	5’GTATGGCATGATAGCGCCCGGAAGAGAGTCAATTCAGGGTGGTGAATGTGAAAAAAAAAAACCAGTAACGTTATACGATG3’	recombineering of A_{10}*lacI* (left)
**OC365**	5'TAATGCAGCTGGCACGACAGGTTTCCCGACTGGAAAGCGGGCAGTGAGCGGTGTAGGCTGGAGCTGCTTC3’	*lacI*-*lacZ* fusion/ΔP*lac*::*knR* (left)
**OC366**	5’CGTTGTAAAACGACGGCCAGTGAATCCGTAATCATGGTCATAGCTGTTTCCTGCCTTAGTTCCTATTCCGAAG3’	*lacI*-*lacZ* fusion/ΔP*lac*::*knR* (right)
**OC464**	5’AGATCGCACTCCAGCCAGC3’	recombineering with OC282, 283, 359 and 360 (right)
