# Supplementary material for: Heritable Change Caused by Transient Transcription Errors
Source: PLoS Genet. 2013 Jun 27;9(6):e1003595. doi: 10.1371/journal.pgen.1003595 (PMC3694819; doi:10.1371/journal.pgen.1003595)
Supplement: Text S1 — Considering lacI +→lacI − forward mutation frequency analysis. (DOCX) [file pgen.1003595.s012.docx]

The *lacI* gene is a well studied mutational target [33,67], and references therein. The spontaneous mutational spectrum is dominated by a frameshift hotspot at the wild-type sequence (CTGG)_3_ with fully 70% of all spontaneous mutation being due to the addition or loss of a CTGG repeat [68-70]. The *lacI*^+^ → *lacI*^-^ forward mutation frequency is on the order of 2-3.5 x 10^-6^ [68-71]. Importantly, when the (CTGG)_3_ frameshift hotspot has been disabled (site-specific mutagenesis to destroy the triple repeat, but maintain the native coding potential), the spontaneous mutation frequency is still on the order of 1.3 x 10^-6^ [71], demonstrating the open robust nature of the *lacI* forward mutational target. In other words, when one goes from a situation in which no frameshift events are observed at this site (the non-hotspot *lacI* gene) to one in which fully 70% of all mutations are frameshift events at this site, the spontaneous mutation frequency only increases about 2.5-fold. Therefore, although we did create an A_9_ run which should be problematic for both RNA and DNA polymerases, it is not surprising that the observed spontaneous mutation frequency is not significantly different between the A_9_ and A_5_GA_3_ *lacI* alleles when one takes into account the open nature of the *lacI* target. It should be noted that the A_9_ and A_5_GA_3_ sequences were appended to a wild-type *lacI* gene that does include the potent (CTGG)_3_ frameshift hotspot. One straightforward explanation is that the (CTGG)_3_ frameshift hotspot remains the dominant mutational site in the A_9_ and A_5_GA_3_ *lacI* alleles. Moreover, while it has been amply demonstrated that this (CTGG)_3_ site is problematic for DNA polymerases, there is no information on how RNA polymerases deal with this site. Indeed, if this site is transcribed efficiently by RNA polymerase, that may also explain the significant increase in epigenetic switch frequency but no increase in mutation frequency between the A_9_ and A_5_GA_3_ strains, since the increase in epigenetic switching due to the A_9_ run will not be overshadowed by transcriptional events at the (CTGG)_3_ frameshift hotspot.
