# Supplementary material for: Heritable Change Caused by Transient Transcription Errors
Source: PLoS Genet. 2013 Jun 27;9(6):e1003595. doi: 10.1371/journal.pgen.1003595 (PMC3694819; doi:10.1371/journal.pgen.1003595)
Supplement: Text S2 — References for supporting information. (DOCX) [file pgen.1003595.s013.docx]

Text S2. References for Supporting Information

66. Baba T, Ara T, Hasegawa M, Takai Y, Okumura Y, et al. (2006) Construction of *Escherichia coli* K-12 in-frame, single-gene knockout mutants: the Keio collection. Mol Syst Biol 2. doi:10.1038/msb4100050.

67. Gordon AJE, Burns PA, Fix DF, Yatagai F, Allen FL, et al. (1988) Missense mutation in the *lacI* gene of *Escherichia coli*: Inferences on the structure of the repressor protein. J Mol Biol 200: 239–251.

68. Farabaugh PJ, Schmeissner U, Hofer M, Miller JH (1978) Genetic studies of the *lac* repressor. VII. On the molecular nature of spontaneous hotspots in the *lacI* gene of *Escherichia coli*. J Mol Biol 126: 847–857.

69. Schaaper RM, Danforth BN, Glickman BW (1986) Mechanisms of spontaneous mutagenesis: an analysis of the spectrum of spontaneous mutation in the *Escherichia coli lacI* gene. J Mol Biol 189: 273–284.

70. Halliday JA, Glickman BW (1991) Mechanisms of spontaneous mutation in DNA repair-proficient *Escherichia coli*. Mutat Res 250: 55–71.

71. Schaaper RM, Dunn RL (1991) Spontaneous mutation in the *Escherichia coli lacI* gene. Genetics 129: 317–326.
